# Dramatic Enhancement in Polylactide Hydrolysis and Biodegradability Utilizing Low Levels of Organic Anhydrides As Masked Acids

**DOI:** 10.1021/acscentsci.6c00395

**Published:** 2026-06-17

**Authors:** Jinsol Yook, Eric D. Rachita, Naba K. Kalita, Christopher J. Ellison, Marc A. Hillmyer

**Affiliations:** † Department of Chemistry, 5635 University of Minnesota, Minneapolis, Minnesota 55455, United States; ‡ Department of Chemical Engineering and Materials Science, University of Minnesota, Minneapolis, Minnesota 55455, United States; § Natural Resources Research Institute, University of Minnesota, Duluth, Minnesota 55811, United States

## Abstract

Polylactide (PLA) is the world’s top synthetic biopolymer and a major contributor to the circular plastic economy by virtue of its biodegradation and full biomineralization into benign end products. Nevertheless, efficient and complete degradation of PLA at its end of use remains constrained by the need for engineered industrial composting environments that operate at elevated temperatures and high humidity levels. In this work, PLA samples containing low levels of phthalic or 2-sulfobenzoic acid anhydride additives were prepared using industrially viable melt-processing. These blends retained mechanical properties nearly identical to that of neat PLA while exhibiting significantly accelerated hydrolytic degradation, achieving appreciable mass losses within one month at 50 °C in artificial seawater. Remarkably, even trace (0.01 wt %, 100 ppm) incorporation of 2-sulfobenzoic acid cyclic anhydride proved highly effective in promoting PLA hydrolysis even at temperatures below the glass transition temperature of the parent material. Furthermore, incorporation of 2-sulfobenzoic acid cyclic anhydride (0.1 wt %) enabled rapid biodegradation under composting conditions at 58 °C, achieving 90% within 11 days, surpassing that of neat PLA. Using anhydrides as strong acid-generating additives enables acceleration of PLA hydrolysis and biodegradation without compromising its other attractive properties, thereby opening a viable pathway toward realizing the full potential of PLA in a larger array of applications.

## Graphical abstract

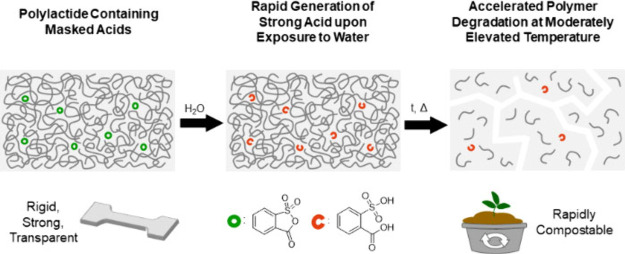

## Introduction

Plastics have become indispensable in modern life, by virtue of their chemical stability, low density, outstanding performance in myriad applications, and low cost.^1^ As a result, global polymer production has reached nearly half a billion metric tons (Mt), a colossal amount that is projected to double within the next two decades.^2,3^ Widescale plastic proliferation has precipitated the relentless accumulation of pernicious waste that has been exacerbated by low recycling rates for most plastics, limited waste management infrastructure, and the intrinsic resistance of nearly all plastics to environmental degradation.^4,5^ To combat this challenge, compostable plastics are re-emerging as viable alternatives to traditional recalcitrant plastics given their propensity to biomineralize into innocuous carbon dioxide, water, and biomass.^6^ Such ephemeral plastics will play an important role in a sustainable plastic future when economic and other practical considerations are effectively addressed.

Polylactide (PLA) is one of the most prominent compostable polymers, accounting for two-thirds of total biobased and biodegradable plastics production.^7^ Its favorable characteristics—biodegradability, biocompatibility, mechanical strength, processability, and optical transparency—have facilitated adoption in, for example, food packaging, biomedical devices, and textiles.^8,9^ Nevertheless, efficient biodegradation of PLA occurs only under the controlled conditions typically found in engineered environments provided by industrial composting facilities that operate at elevated temperatures (55–75 °C) and sufficient humidity (40–65%).^10–12^ Under typical ambient conditions (∼20 °C, ∼50% relative humidity), estimates suggest that high molar mass PLA will persist for over a century, with only modest molar mass reductions.^13,14^ Unfortunately, only 18% of the U.S. population has access to industrial composting infrastructure. This situation must be addressed to help prevent PLA products from ending up in landfills or the environment.^15,16^

To enhance the degradability of PLA, various strategies have been pursued such as blending PLA with more rapidly degrading polymers and introducing cleavable linkages using copolymerization strategies.^17–20^ Blending typically requires a high additive loading (>10 wt %), which can significantly alter the intrinsic properties of PLA and often compromise its desirable mechanical, thermal, and/or optical properties.^17,21^ As an example of a copolymerization strategy, we recently developed a salicylate-containing cyclic monomer, salicylic methyl glycolide (SMG), and incorporated it into the backbone of PLA via transesterification.^22,23^ The resulting copolymers exhibited accelerated degradation, attributed to the release of salicylic acid upon hydrolysis; however, the requirement of high SMG content (7–25 mol %) and the multistep, relatively low efficiency monomer synthesis motivated us to explore much more practical approaches that included salicylic acid. To that end, we recently demonstrated that direct melt blending of salicylic acid into PLA substantially enhances PLA degradation with additive contents ≤ 2 wt %, validating the feasibility of this small molecule blending approach.^24^

Unfortunately, directly incorporating acids can lead to hydrolysis of PLA during melt processing in the presence of adventitious water, thereby reducing PLA molar mass and mechanical strength.^25,26^ In this work, we employ a strategy that utilizes commercially available anhydrides, phthalic anhydride (PAn) and 2-sulfobenzoic acid cyclic anhydride (SAn), that function as protected or masked acids that we posited would be more compatible with standard PLA melt processing protocols. Upon hydrolysis, these anhydrides generate strong acids (p*K*_a_: about 3 for phthalic acid and about −1 for 2-sulfobenzoic acid)^27,28^ for which safety profiles have been reported.^29–31^ We expect that these strong acids can catalyze the hydrolytic degradation of PLA under a broader array of biodegradation conditions, thus expanding practical end of use options for PLA products.

## Results and Discussion

Commercially available amorphous PLA (Natureworks 4060D) was melt-blended with PAn and SAn using a twin-screw compounder, followed by melt compression to produce films with a uniform thickness ≈ 0.33 mm (Figure 1). The melting temperatures of PAn (131 °C) and SAn (116 °C) are lower than the PLA processing temperatures (160 and 140 °C), which facilitated mixing of these liquids with the viscoelastic PLA at these blending temperatures. The blends are as transparent as neat PLA (Figures 1 and S1) and denoted as PAn# and SAn#, where # represents the anhydride loading as a mass percentage of the entire blend.

**Figure 1:**
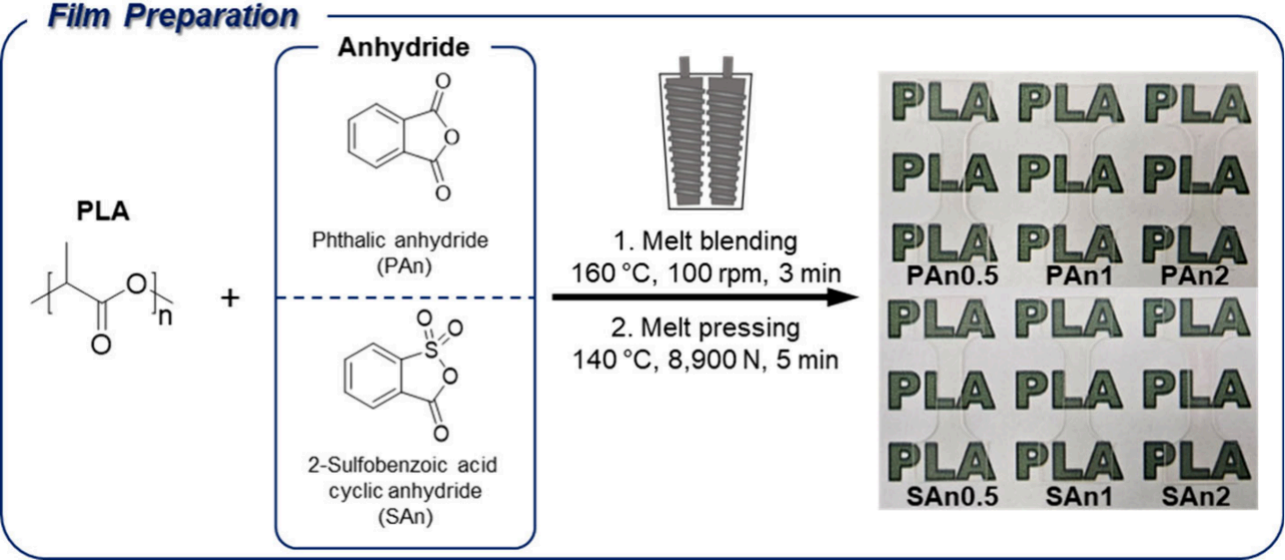
Preparation procedure for neat PLA and anhydride-containing blends, and photographs of representative tensile bars.

Detailed characterization data for the base PLA and its blends are presented in the Supporting Information (Figures S2–S6 and Tables S2–S4). We verified the anhydride content in blends using the aromatic proton signals in the ^1^H NMR spectra (Figure S2). The size exclusion chromatography (SEC) data for the anhydride-containing blends closely overlapped with that of neat PLA (Figure S3). The number-average molar mass (*M*_n_) of PLA was 72 kg mol^–1^, and that of PAn# samples ranged from 68 to 71 kg mol^–1^, corresponding to an insignificant reduction relative to neat PLA (Table S2). Modest *M*_n_ decreases (55–65 kg mol^–1^) were observed for the SAn# blends. In contrast, direct incorporation of 1 wt % of 2-sulfobenzoic acid (the hydrolysis product of SAn) led to substantial PLA degradation during melt processing and gave *M*_n_ = 37 kg mol^–1^, which resulted in severe deterioration of the PLA blend mechanical properties (Figure S4 and Table S3). All PLA blends containing anhydride additives exhibited mechanical properties comparable to those of neat PLA, suggesting the feasibility of incorporating these additives using conventional melt processing for the fabrication of PLA blends (Figure S5 and Table S4). Differential scanning calorimetry (DSC) analysis revealed that amorphous PLA exhibited a glass transition temperature (*T*_g_) of 55 °C, which decreased a few degrees with increasing anhydride content to as low as 50 °C at 2 wt % additive loadings (Figure S6). This *T*_g_ reduction suggests a modest plasticizing effect of PAn and SAn, consistent with other related additives.^32^

Hydrolytic degradation of the parent PLA and blends was monitored for one month at 50 °C in artificial seawater (pH ≈ 8.0). The elevated temperature (50 °C ≤ *T*_g_) was selected to provide preliminary assessment of anhydride effectiveness under accelerated testing conditions. Figure 2 presents the normalized mass profiles of PLA, PAn#, and SAn# as a function of hydrolysis time. PLA showed a slight mass increase (∼1.4 wt %) within 3 days, corresponding to its expected equilibrium water content at this temperature.^33^ Over the ensuing month, PLA exhibited a small additional overall mass change, showing only a minor increase up to 2.3 wt %. Conversely, both PAn# and SAn# underwent substantial mass loss that increased with anhydride content. After 31 days, the fractional remaining mass decreased to 0.74–0.53 for PAn# and 0.58–0.51 for SAn#, depending on loading levels. Like PLA, both blends exhibited a mass increase at very early stages in the degradation experiment due to water uptake. This increase was followed by steady mass loss, characteristic of bulk erosion where water diffusion outpaces hydrolytic chain scission.^34^ Unlike the PLA and PAn# samples, SAn# samples showed rapid and substantial mass increase with peak mass increases as high as 48% for SAn2. This remarkable early time water absorption is likely due to the hygroscopic nature of SAn upon rapid hydrolysis to give 2-sulfobenzoic acid (Figure S7 and Table S5). However, the incorporation of SAn showed negligible impact on the initial water diffusion in the polymer matrices confirmed by similar diffusion coefficients for SAn0.5 (0.62 × 10^–8^ cm^2^ s^–1^) and PLA (0.61 × 10^–8^ cm^2^ s^–1^) at ambient temperature (Figure S8). Scanning electron microscope (SEM) images revealed that a representative SAn1 sample developed a nanoporous surface after 3 days, whereas PAn1 remained comparatively smooth (Figure S9). Cross-sectional SEM images of SAn1 further confirmed the well-developed nanoporous structure. The nanoscopic pores in SAn1 likely originated from localized hydrolysis accelerated by highly acidic 2-sulfobenzoic acid, which promoted PLA degradation. The generation of more carboxylic acid from PLA backbone ester hydrolysis, and concomitant increases in water absorption, further accelerated hydrolysis. Acid formation was observed using PLA blends containing bromophenol blue as a pH indicator, which exhibited a pronounced yellow coloration over time indicative of a low pH environment (Figure S10). This autocatalytic aspect of PAn# and SAn# blends results in very facile overall degradation compared to the PLA control at as little as 0.5 wt % loading.

**Figure 2:**
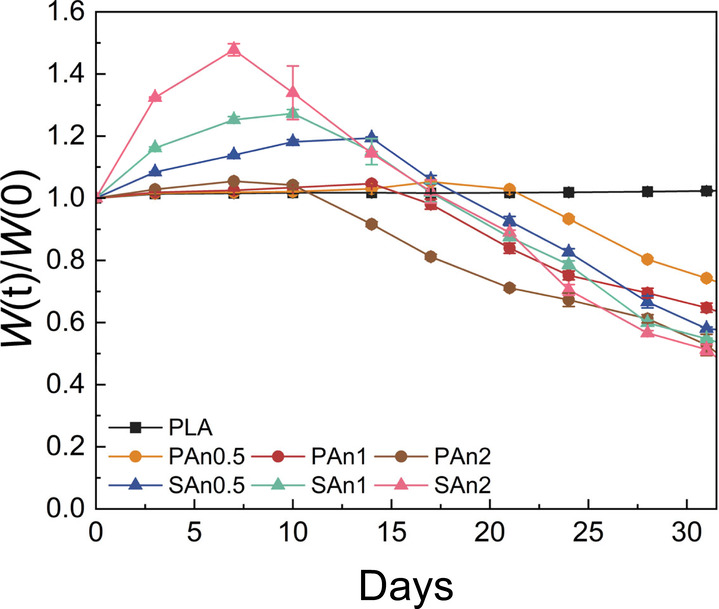
Fractional mass of neat PLA, PAn#, and SAn# during hydrolytic degradation at 50 °C in artificial seawater for one month.

Figure S11 presents the SEC traces of the insoluble fractions of the blends recovered from the hydrolysis experiment. PLA exhibited a gradual peak shift toward higher elution times, indicating a continuous molar mass decrease despite negligible mass change. Both the PAn# and SAn# samples displayed much more pronounced molar mass reductions over the duration of the experiment, with higher additive contents resulting in faster molar mass reductions. In addition, their SEC profiles evolved from unimodal to bimodal, suggesting an inhomogeneous degradation process, likely associated with progressive polylactide crystallization during hydrolysis.^35^ Notably, SAn# samples underwent a drastic molar mass decrease within only 3 days, reaching ∼20 kg mol^–1^ or lower, whereas PLA and PAn# required approximately 21 and about 7–10 days, respectively, underscoring the superior efficiency of SAn in accelerating PLA degradation.

^1^H NMR analysis further confirmed the molar mass changes in PLA, PAn#, and SAn# (Figures S12–S18). The relative intensity of the methine end-group signal (4.2 ppm) to that of polylactide repeating units (5.1–5.3 ppm) increased over time in all samples, indicating progressive chain scission. Figure S19a summarizes the molar mass determined from these NMR signals as a function of hydrolysis time. PLA exhibited an exponential decrease in molar mass, and all blends displayed distinctly faster molar mass reductions, consistent with the SEC results. In the blends, the molar mass gradually decreased until reaching around 2 kg mol^–1^, after which it leveled off. This plateau arose because polylactide oligomers with molar masses ≈ 1 kg mol^–1^ or smaller are water-soluble and continued to leach into the aqueous solution.^36^ NMR evidence (Figure S20) showed that SAn in the blends was completely hydrolyzed within 3 days. In contrast, PAn in the blends required 7 days for complete hydrolysis, with intact PAn remaining after 3 days (28% in PAn0.5, 8.8% in PAn1, and 4.1% in PAn2) (Figure S19b). The additive content remained nearly constant at the early stages but gradually decreased thereafter, coinciding with the onset of sample mass loss and thus attributed to the loss of water-soluble small molecules from the solid samples.

The ^1^H NMR spectra also revealed the formation of lactic acid during PLA hydrolysis, as confirmed by the appearance of a new signal at 4.04 ppm corresponding to the methine of lactic acid (Figure S12). In PLA, this signal appeared after 28 days, whereas it emerged much earlier in PAn#, between 7 and 14 days, depending on the PAn content. Remarkably, all SAn# samples generated a substantial amount of lactic acid within only 3 days (Figure 3). The maximum lactic acid content increased from 2.3 to 4.9 wt % with higher SAn content, corresponding to 4–10 times that of PAn# (0.47–0.58 wt %) and 18–38 times that of PLA (0.13 wt % at 28 days). After reaching its maximum, the lactic acid content gradually decreased as it dissolved in the aqueous media. This distinctive behavior in the SAn# samples demonstrates a dramatically accelerated, acid-catalyzed hydrolysis driven by synergistic interplay of extensive water uptake and the highly acidic 2-sulfobenzoic acid.

**Figure 3:**
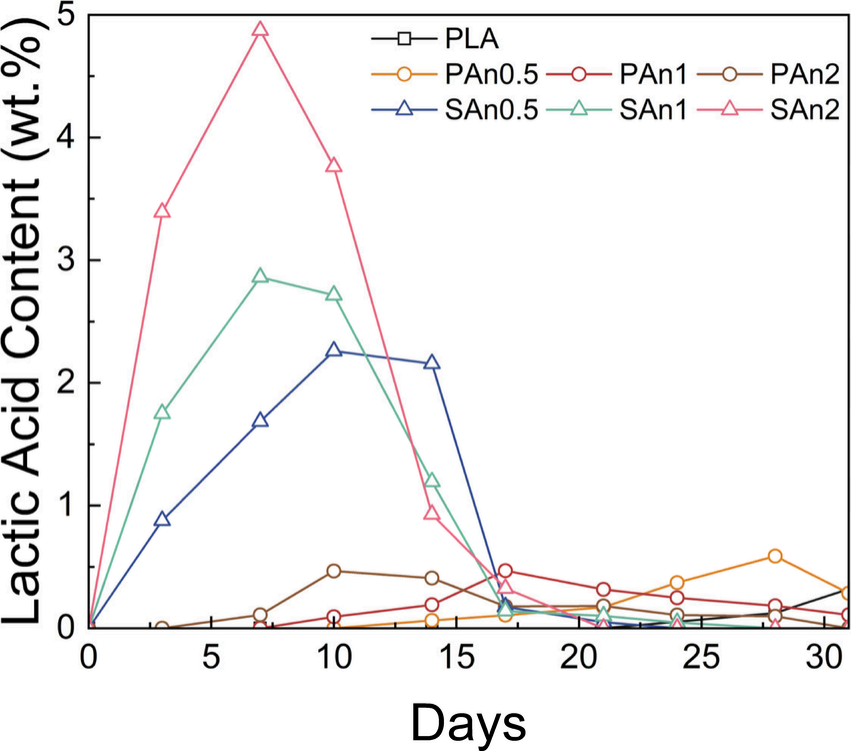
Content of lactic acid generated during hydrolytic degradation at 50 °C in neat PLA, PAn#, and SAn# determined by ^1^H NMR.

Given the remarkable enhancement of polylactide hydrolysis in the SAn# blends, degradation experiments of neat PLA and SAn0.5 were further conducted over a range of temperatures (45, 50, 55, and 65 °C) and longer time frames (Figure 4). This temperature range corresponds to ideal home composting conditions (45–65 °C).^37^ At all temperatures, PLA showed a similar mass profile characterized by an initial small increase due to water uptake followed by mass loss due to oligomer dissolution over longer times, and the overall degradation rate significantly decreased at lower temperatures. PLA underwent nearly complete mass loss within 28 days at 65 °C, where the final data point in the fractional mass profile represents the last measurable mass of the sample prior to its disappearance. In contrast, substantially longer periods of 56, 77, and 147 days were required at 55, 50, and 45 °C, respectively. Notably, the degradation rate slowed considerably at 45 °C, taking about 70 days longer than at 50 °C, while the difference between 50 and 55 °C was relatively small (21 days). These results confirm the strong temperature dependence of PLA degradation and the significant difference in behavior for degradation temperatures below *T*_g_ due to restricted water diffusion.^38,39^ SAn0.5 followed a similar temperature-dependent trend but consistently degraded much faster than PLA at all temperatures. Complete mass loss of SAn0.5 occurred within 98, 56, 42, and 28 days at 45, 50, 55, and 65 °C, respectively. Comparatively, this is 49, 21, and 14 days earlier than neat PLA at 45, 50, and 55 °C, respectively. This difference was particularly large at 45 °C, suggesting that incorporation of SAn effectively promoted a rapid initial decrease in molar mass, thereby mitigating the reduced chain mobility that limits PLA degradation below *T*_g_ and ultimately enhancing the overall degradation.

**Figure 4:**
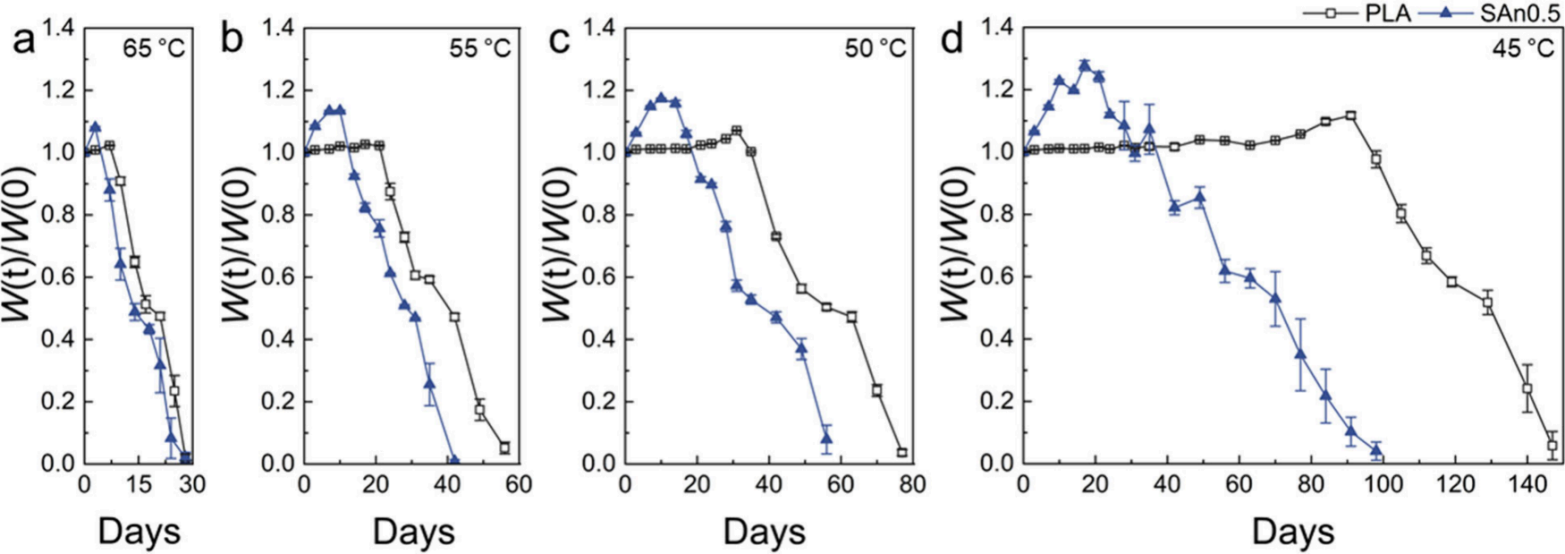
Fractional mass of PLA and SAn0.5 during hydrolytic degradation at (a) 65 °C, (b) 55 °C, (c) 50 °C, and (d) 45 °C in artificial seawater.

The SEC results further corroborate the temperature-dependent degradation behavior (Figures S21 and S22). For PLA, the decrease in *M*_n_ became progressively slower with decreasing temperature and was significantly suppressed at 45 °C, consistent with the trend observed in mass loss profiles (Figure S23). The SAn0.5 samples exhibited a much faster *M*_n_ decrease, reaching ∼20 kg mol^–1^ or lower within 3 days at all temperatures. In addition, all samples at all temperatures showed a transition from unimodal to bimodal distribution, consistent with our observations from the previous SEC results.

^1^H NMR spectroscopy results further validated the temperature-dependent molar mass decrease trends observed using SEC analysis (Figures S24–S32). SAn0.5 exhibited a faster decrease in molar mass than PLA at all temperatures. SAn0.5 showed 1.8–5.3 times higher hydrolysis rate constants than that of PLA at 50 °C (Table S6). This represents a greater enhancement compared to our previous work, where PLA containing 1 wt % salicylic acid showed a 1.2-fold increase in the rate constant relative to neat PLA.^24^ In addition, SAn0.5 produced a substantial amount of lactic acid within 3 days at all temperatures, whereas PLA exhibited a distinct induction period, lasting up to one month at 45 °C (Figure S33). In SAn0.5, the decline in lactic acid content and the point at which it became undetectable occurred earlier at the higher temperature, as the molar mass rapidly reduced to a critical value below which water-soluble molecules could no longer be retained in the degrading sample. The additive content showed a similar temperature-dependent trend. This premature depletion of acids at the higher temperatures may contribute to the less pronounced acceleration of PLA degradation for SAn0.5.

The DSC first heating curves provided complementary insight. Initially, amorphous PLA displayed only a glass transition, which gradually shifted to lower temperatures as *M*_n_ decreased during hydrolysis (Figure S34). An endothermic melting peak emerged during hydrolysis at all temperatures, with crystallization initiating earlier at the higher degradation testing temperature. The endothermic peak became sharper and more intense over time, indicating a narrower crystallite size distribution, improved crystalline order, and increase in crystallinity (*X*_c_). *X*_c_ rose steadily until reaching a plateau between 50–60% across all conditions, attributed to preferential degradation of amorphous domains and progressive development of crystalline regions (Figure S35), as we reported previously.^24^ The melting temperature (*T*_m_) of the PLA in these samples increased gradually to a maximum before decreasing at later stages, suggesting subsequent degradation of crystalline domains. SAn0.5 showed similar DSC trends but underwent faster crystallization due to its more rapid molar mass decrease (Figure S36). This progressive crystallization in both PLA and SAn0.5 during hydrolysis likely contributed to both the bimodal transition in SEC profiles and the deceleration observed during mass loss, the latter of which was reported previously.^24^

Encouraged by the striking efficiency of SAn in accelerating polylactide hydrolysis, we were motivated to further minimize the anhydride content in the SAn# blends. Thus, we explored the hydrolytic degradation behavior at 45 °C in blends containing 0.05 and 0.01 wt % (SAn0.05 and SAn0.01), which correspond to 500 and 100 ppm levels of additive, respectively. These blends exhibited mechanical properties that were comparable to those of the PLA control. Moreover, the *T*_g_ values of these two blends were only 0.3 or 0.6 °C lower than the PLA control (*T*_g_ = 55.4 °C) (Figures S37–S39 and Table S7). Figure 5 unequivocally demonstrates that even these trace levels of SAn incorporation effectively promoted polylactide degradation compared to similarly processed neat material. Consequently, SAn0.01 and SAn0.05 achieved complete mass loss approximately 45 to 24 days earlier than PLA, respectively. Importantly, and in contrast to the sample with higher loading SAn0.5, neither SAn0.01 nor SAn0.05 exhibited massive early time water uptake.

**Figure 5:**
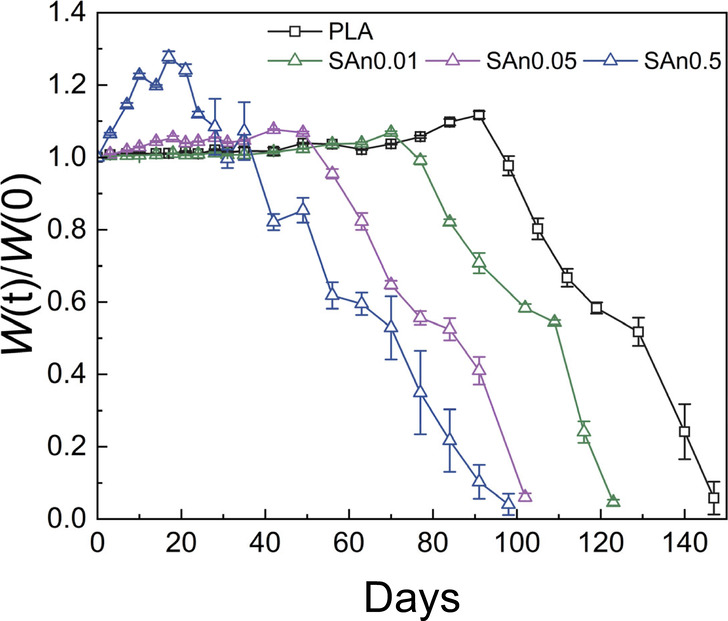
Fractional mass of neat PLA and SAn# with minimal loading of 0.01–0.5 wt % during hydrolytic degradation at 45 °C in artificial seawater.

Instead, their initial water uptake was comparable to that of neat PLA at both room temperature and 50 °C under 95% relative humidity (Figure S40). However, the mass increase at later times scaled with SAn content. This result indicates that minimal SAn contents did not affect the initial water uptake yet remained highly effective in accelerating polylactide degradation, which subsequently promoted water absorption. Both SEC and NMR analyses further supported the accelerated degradation behavior in these blends with ultralow SAn content (Figures S41–S44).

Hydrolytic degradation was also conducted in distilled water at ambient temperature (20–25 °C) (Figure S45). Under these conditions, both PLA and PAn1 blend exhibited negligible mass change over 553 days. While SAn0.01 did not show any significant mass change until 493 days, SAn1 exhibited nearly 50% mass loss after 553 days. Despite the significantly slower degradation rate at ambient temperature, these results demonstrate that the degradation rate can be effectively tuned by varying the additive loading, under ambient aqueous conditions.

To explore the impact of SAn on the properties of the blends over long times, PLA, SAn0.01, and SAn0.05 were evaluated after being stored at ambient conditions (temperature: 20–25 °C, relative humidity: 25–50%) for 11 months. PLA and SAn0.01 exhibited no significant molar mass decrease over this period, as confirmed by negligible shift in SEC profiles (Figure S46). SAn0.05 displayed a peak shift, corresponding to the molar mass reduction to as low as 44 kg mol^–1^ (36% decrease relative to the initial *M*_n_ of 69 kg mol^–1^). The mechanical properties of PLA, SAn0.01, and SAn0.05 were also evaluated (Figure S47 and Table S8). Despite the *M*_n_ reduction observed for SAn0.05, both SAn0.01 and SAn0.05 retained mechanical properties comparable to those of the neat PLA control. These results suggest that the shelf life stability of PLA is largely preserved in the SAn0.05 and SAn0.01 blends containing ultralow SAn contents. In addition, the samples were subjected to cyclic conditioning by storing under ambient conditions for 3 days followed by exposure to humid conditions (temperature: 22–25 °C, relative humidity: 60–80%) for 3 days. Even after two repeated cycles, both SAn0.01 and SAn0.05 maintained mechanical properties comparable to those of neat PLA (Figure S48 and Table S9).

To confirm the industrial compostability of polylactide blends with SAn, we performed respirometry analysis using SAn0.1 as a representative sample and polyethylene terephthalate (PET) as a negative control (Figure S49). The higher additive loading was selected to provide a clearer evaluation of the effect of SAn on biodegradation. Cellulose, a positive control, reached 90% absolute biodegradation (conversion to CO_2_) after 62 days at 58 °C, while the PLA control reached 83% absolute biodegradation over 90 days. In sharp contrast, SAn0.1 exhibited remarkably accelerated biodegradation under these conditions, attaining 90% CO_2_ production within just 11 days and complete biodegradation within 21 days. During biodegradation, the fragmentation of PLA can lead to the release of acidic degradation products, including lactic acid and 2-sulfobenzoic acid. The accumulation of acidic species in compost could potentially lower local pH and inhibit microbial activity; however, this behavior was not observed in this system, as confirmed by the slightly basic final compost (pH 8.6). This could be attributed to the inherent biodegradability of both lactic acid^40^ and 2-sulfobenzoic acid.^41,42^ These results indicate that minimal SAn incorporation promotes complete compostability over a much shorter time frame than PLA, significantly enhancing the overall biodegradation rate without inhibiting microbial activity.

## Conclusions

We have demonstrated the remarkable effectiveness of incorporating anhydrides as latent organic acid generators to accelerate PLA degradation without compromising its intrinsic mechanical and thermal properties both upon preparation and after long-term storage. Notably, SAn exhibited superior efficiency, promoting PLA degradation even at trace levels as low as 100 ppm and under degradation conditions in water well below the *T*_g_ of the material. Moreover, SAn remarkably enhanced the biodegradation of PLA under industrial composting conditions. These findings open new avenues for the design of highly efficient PLA degradation promoters, potentially enabling degradation under mild conditions such as home composting or natural environmental settings and paving the way toward realizing the full potential of PLA in an even broader array of sustainable applications.

## Supplementary Material



## Data Availability

The data used in this study are openly available in the Data Repository for University of Minnesota (DRUM) at https://hdl.handle.net/11299/166578.

## Notes

Materials and methods; characterizations; ^1^H NMR spectra, SEC traces, DSC curves, and tensile test results of as-prepared films; normalized mass, ^1^H NMR spectra, SEC results, and DSC results of PLA and anhydride-containing blends during hydrolysis; shelf life stability test; and respirometry data (PDF)
