# Supplementary material for: Dramatic Enhancement in Polylactide Hydrolysis and Biodegradability Utilizing Low Levels of Organic Anhydrides As Masked Acids
Source: ACS Cent Sci. 2026 Jun 17;12(7):965–71. doi: 10.1021/acscentsci.6c00395 (PMC13397435; doi:10.1021/acscentsci.6c00395)
Supplement: Supplementary file 1 [file oc6c00395_si_001.pdf]

Supporting Information for

Dramatic enhancement in polylactide hydrolytic- and bio-degradability utilizing low levels of organic anhydrides as masked acids

Jinsol Yook[†], Eric D. Rachita,[‡] Naba K. Kalita,[§] Christopher J. Ellison,[‡,*] and Marc A. Hillmyer[†,*]

[†]Department of Chemistry, University of Minnesota, Minneapolis, MN 55455, United States

[‡]Department of Chemical Engineering and Materials Science, University of Minnesota, Minneapolis, MN 55455, United States

[§]Natural Resources Research Institute, University of Minnesota Duluth, Duluth, MN 55811, United States

*Corresponding authors (e-mail: cellison@umn.edu, hillmyer@umn.edu)

Materials and Methods

Materials. Phthalic anhydride (PAn, MilliporeSigma), 2-sulfobenzoic acid cyclic anhydride (SAn, Santa Cruz Biotechnology), 2-sulfobenzoic acid (SAcid, Fisher Scientific), pyromellitic dianhydride (PDAn, MilliporeSigma), and amorphous polylactide (PLA, NatureWorks, 4060D) were purchased and dried under dynamic vacuum at 40 °C overnight prior to usage. Amorphous PLA 4060D was selected as a representative PLA material to investigate the effect of additives while minimizing confounding effects arising from the differences in crystallinity. Artificial seawater was prepared by dissolving salts with specified compositions following ASTM D1141-98 into distilled water. [1]

Film preparation. PLA 4060D pellets were added into a 15 cc twin-screw extruder (Xplore MC15) with the appropriate anhydride loading, followed by mixing at 160 °C with 100 rpm screw speed for 3 min. The extruded materials were placed between two Teflon sheets, and placed on a hydraulic press (Wabash Genesis) preheated to 140 °C. The materials were annealed at 140 °C for 5 min without applying any pressure and pressed with 2,000 lbs of force (approximately 8,900 N) for another 5 min at the same temperature. The thickness of melt-pressed films was around 0.33 mm.

Hydrolytic degradation experiment. The melt-pressed films were punched using a hole punch to prepare disks with 6 mm diameter and the mass of each disk was in the range of 12–15 mg. This procedure was adopted to exclude the effect of the specimen's geometric factors on degradation behavior, as shape, dimensions, and surface area have been known to significantly affect the degradation behavior. [2] Three disks were placed into a 20 mL vial with artificial seawater, maintaining a solid concentration of 5 mg mL^{-1}, and then placed into a HERAtherm OMH60-S convection oven set to 50 °C. The water insoluble parts were periodically retrieved, dried at

ambient conditions to remove surface moisture, and weighed to investigate the mass loss over time. The liquid was replaced with fresh artificial seawater after each mass measurement to avoid significant pH fluctuations and to better simulate environmentally relevant conditions. Once the disks lost structural integrity, the remaining insoluble materials were collected by filtering the mixture through a paper filter (Fischer Scientific, P8 grade) with a particle retention between 20-25 μm, followed by drying under ambient conditions before measuring the mass. Degradation experiments at other temperatures (45 °C, 55 °C, and 65 °C) were conducted following the same procedure, except that the samples were placed in a water bath set to the desired temperature. The temperature was verified using a H-B Instrument SP Scienceware calibrated electronic thermometer.

Biodegradation experiment. Mature compost (4–6 months old) was collected from an industrial composting facility (Resource Renew, Duluth, MN, USA) processing food waste, yard waste, and seasonal animal manure. The compost was sieved through a 5 mm sieve and adjusted to a moisture content of 45–60% by addition of deionized water, resulting in an inoculum with pH 8.

Biodegradation tests were conducted according to ASTM D5338-15(2021),[3] which specifies aerobic composting at thermophilic temperature (58 ± 2 °C). [4] The experiments were performed using a respirometer (ER/60/1.4, ECHO instruments, Slovenske Konjice, Slovenia) equipped with 2 L composting reactors maintained at 58 ± 2 °C. Microcrystalline cellulose (particle size ~20 μm) was used as the positive control and polyethylene terephthalate (PET, PolyQuest) as the negative control. All polymer samples were cryogenically milled into fine powders (average particle size 180~230 μm). For each test reactor, 20 g of polymer samples were mixed with 240 g of compost inoculum, and all samples including positive and negative controls

were prepared in triplicate. Blank reactors containing only compost (240 g) were also prepared in triplicate to account for background respiration. An air-flow (100 mL min^{-1}) was supplied to each reactor to ensure aerobic conditions. The evolved CO_2 was continuously monitored using the built-in gas sensors to calculate the absolute biodegradation according to equation (1).

$$\text{Absolute biodegradation (\%)} = \left(\frac{mean\ CO_{2,\ sample} - mean\ CO_{2,\ blank}}{sample\ mass \times carbon\ content\ (\%) \times 44/12}\right) \times 100 \qquad (1)$$

The carbon content of each sample was measured using Elementar VarioMAX C/N analyzer and the values are listed in Table S1.

Table S1. Carbon contents of samples

Samples	Carbon (%)
Cellulose	44.4[a]
PLA	50.4
SAn0.1	50.0
PET	62.6

[a]A theoretical carbon content of cellulose[5]

Characterizations

<u>*^{1}H NMR spectroscopy.*</u> ^{1}H nuclear magnetic resonance (NMR) spectra were recorded on a 400 MHz Bruker Advance III HD spectrometer with a SampleXpress autosampler. Samples were dissolved in DMSO-*d*$_6$ at a concentration of 20 mg mL^{-1}. Molar masses of polymers were calculated by end-group analysis based on polylactide repeating unit methine signal at 5.1–5.3 ppm and end group methine signal at 4.2 ppm.

<u>*Size exclusion chromatography (SEC).*</u> SEC was performed on a Waters Arc HPLC system fitted with a Wyatt DAWN 8 multi-angle laser light scattering (MALS) detector and a Wyatt Optilab diffractive refractive index (RI) detector through TSKgel G6000HHR columns using

tetrahydrofuran (THF) as a mobile phase at a flow rate of 1 mL min^{-1}. Samples were dissolved into THF at a concentration of 3 mg mL^{-1} and filtered using a 0.22 μm syringe filter.

Differential scanning calorimetry (DSC). DSC experiments were performed on a TA Instrument Discovery DSC under a nitrogen atmosphere. Before analysis, the degraded samples were dried under dynamic vacuum at 50 °C for 3 h. 2–6 mg of samples were hermetically encapsulated into aluminum Tzero pans. The samples were heated to 180 °C, cooled to 0 °C, and then heated to 180 °C at heating and cooling rates of 10 °C min^{-1} with an additional 3 min for isothermal equilibration at the end of each cycle. The polylactide crystallinity was calculated using the following equation (2):

$$X_c = \frac{\Delta H_f - \Delta H_c}{\Delta H_f^0} \tag{2}$$

where ΔH_f is the enthalpy of fusion and ΔH_c is the enthalpy of crystallization from the first heating scan. ΔH_f^0 is the theoretical enthalpy of fusion for 100% crystalline polylactide at the equilibrium melting temperature (ΔH_f^0 = 93 J g^{-1}). [6]

Uniaxial tensile testing The melt-pressed films were cut into dog-bone shaped specimens with dimensions 0.33 × 5.0 × 20 mm (thickness × gauge width × gauge length). Specimens were placed between flat grips with sandpaper to avoid slipping. The tensile test was performed on a Shimadzu Autograph AGS-X instrument with an extension rate of 5 mm min^{-1}. The ultimate strength, Young's modulus, strain at break, and toughness were calculated based on the stress-strain data. At least five specimens of each sample were tested to calculate average value and standard deviation.

Scanning electron microscopy (SEM) SEM images were collected on a Hitachi SU8230 using a 5 kV accelerating voltage. Samples were dried under dynamic vacuum at room temperature overnight before imaging.

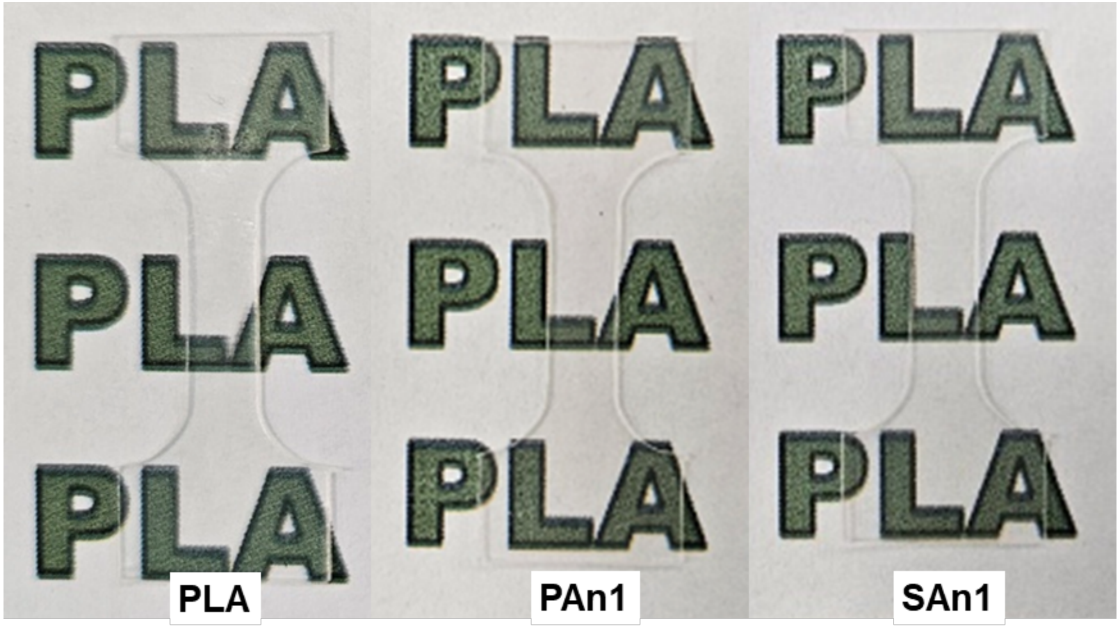

Figure S1. A photograph of as-prepared PLA, PAn1, and SAn1.

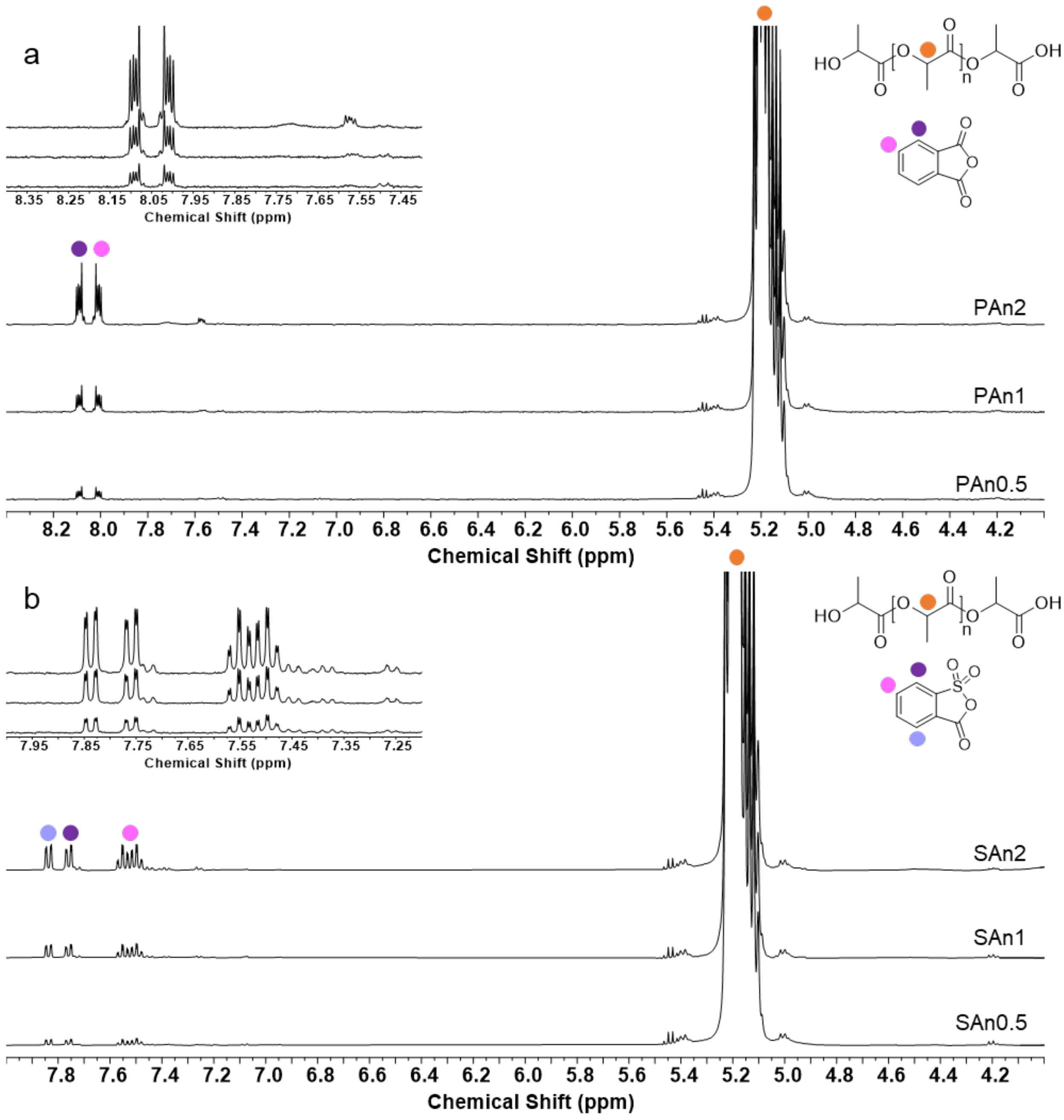

Figure S2. ^{1}H NMR spectra of as-prepared (a) PAn# and (b) SAn# recorded in DMSO-d_6. Insets display the aromatic proton regions. The spectra are normalized to the methine signal of polylactide repeating unit at 5.1–5.3 ppm. The anhydride contents were calculated as 0.39 (PAn0.5), 0.76 (PAn1), 1.72 (PAn2), 0.41 (SAn0.5), 0.84 (SAn1), and 1.59 (SAn2).

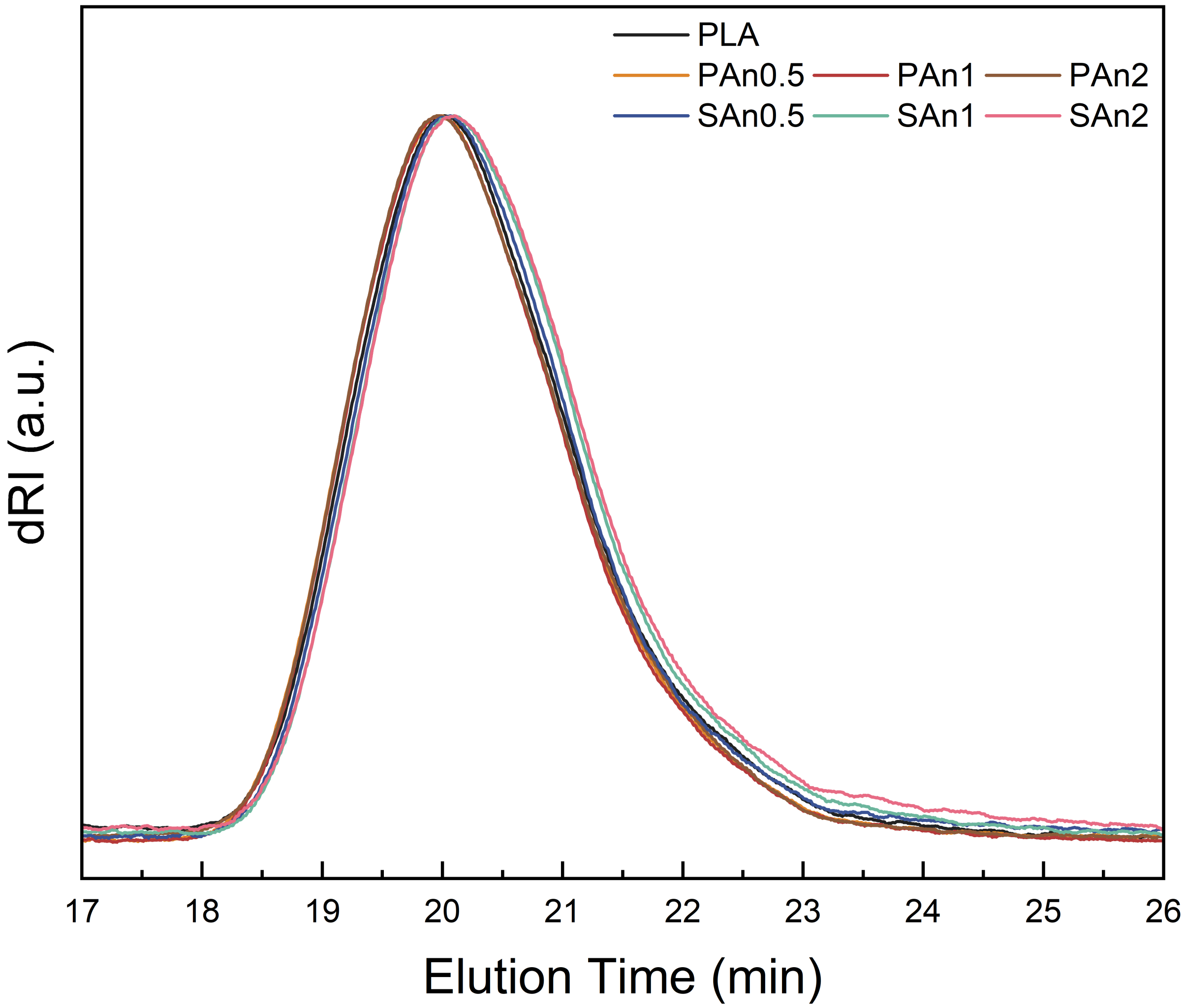

Figure S3. SEC traces of as-prepared PLA, PAn#, and SAn# using THF as an eluent.

Table S2. Molar mass and polydispersity ($Đ$) determined from SEC data.

Samples	M_n (kg mol^{1})	M_w (kg mol^{1})	$Đ^a$
PLA	72.1	131	1.82
PAn0.5	68.2	128	1.88
PAn1	68.4	129	1.89
PAn2	70.9	129	1.82
SAn0.5	64.6	112	1.74
SAn1	55.9	105	1.88
SAn2	54.9	105	1.91

[a] $Đ = M_w/M_n$

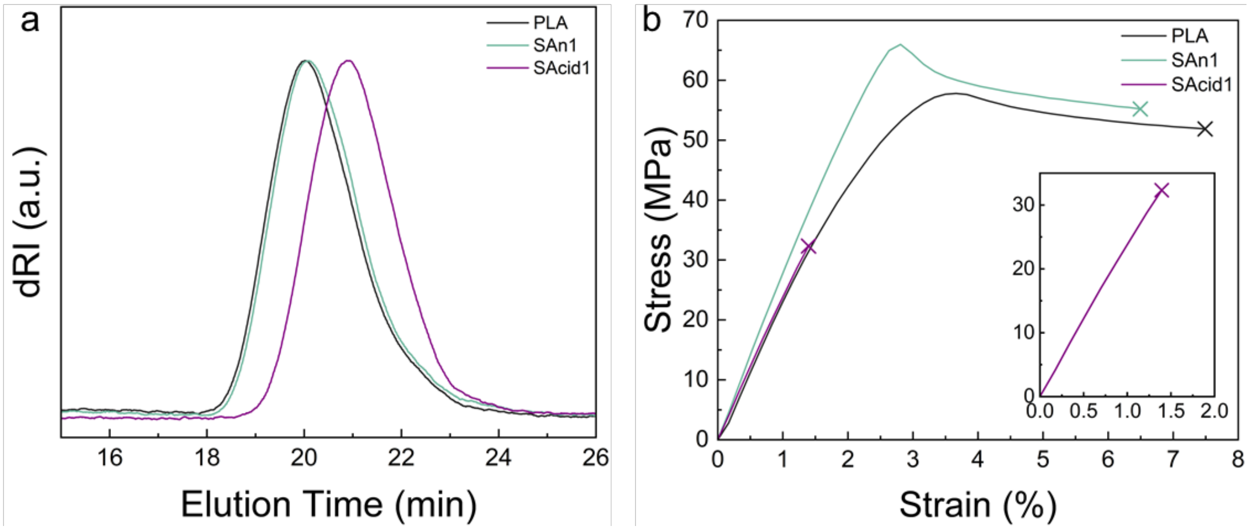

Figure S4. (a) SEC traces of as-prepared PLA, SAn1, and SAcid1. SAcid1 represents a PLA blend containing 1 wt% of 2-sulfobenzoic acid. The corresponding M_n, M_w, and $Đ$ values of SAcid1 are 36.6 kg mol^{-1}, 60.6 kg mol^{-1}, and 1.65, respectively. (b) Representative stress-strain curves of as-prepared PLA, SAn1, and SAcid1, with an inset highlighting the curve of SAcid1.

Table S3. Mechanical properties of PLA, SAn1, and SAcid1.

Sample	Ultimate Strength (MPa)	Young's Modulus (GPa)	Strain at Break (%)	Toughness (MJ m^{-3})
PLA	58.0 ± 0.6	2.5 ± 0.1	6.8 ± 2.0	3.0 ± 1.0
SAn1	62.0 ± 3.2	2.8 ± 0.1	7.3 ± 2.3	3.4 ± 1.2
SAcid1	36.7 ± 6.4	3.2 ± 0.8	1.3 ± 0.1	0.2 ± 0.03

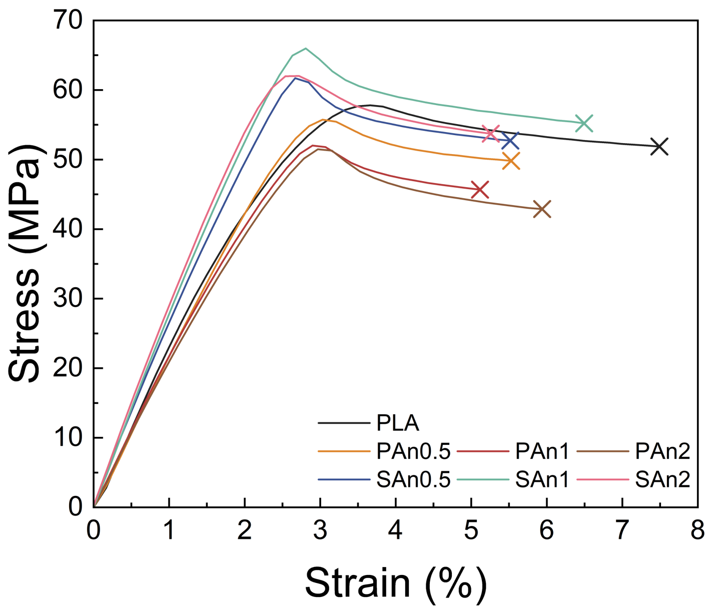

Figure S5. Representative stress-strain curves of as-prepared PLA, PAn#, and SAn# extended at 5 mm min^{-1}. PLA and SAn1 curves are identical with those in Figure S4.

Table S4. Mechanical properties of as-prepared PLA, PAn#, and SAn#.

Sample	Ultimate strength (MPa)	Young's modulus (GPa)	Strain at break (%)	Toughness (MJ m^{-3})
PLA	58.0 ± 0.6	2.5 ± 0.1	6.8 ± 2.0	3.0 ± 1.0
PAn0.5	55.9 ± 2.7	2.3 ± 0.1	5.1 ± 0.7	2.0 ± 0.3
PAn1	52.4 ± 4.0	2.4 ± 0.1	4.8 ± 0.6	1.8 ± 0.3
PAn2	52.2 ± 1.3	2.2 ± 0.1	5.3 ± 0.9	2.0 ± 0.4
SAn0.5	60.9 ± 1.8	2.8 ± 0.04	5.8 ± 0.9	2.6 ± 0.5
SAn1	62.0 ± 3.2	2.8 ± 0.1	7.3 ± 2.3	3.4 ± 1.2
SAn2	62.2 ± 1.4	2.9 ± 0.1	5.5 ± 0.9	2.6 ± 0.5

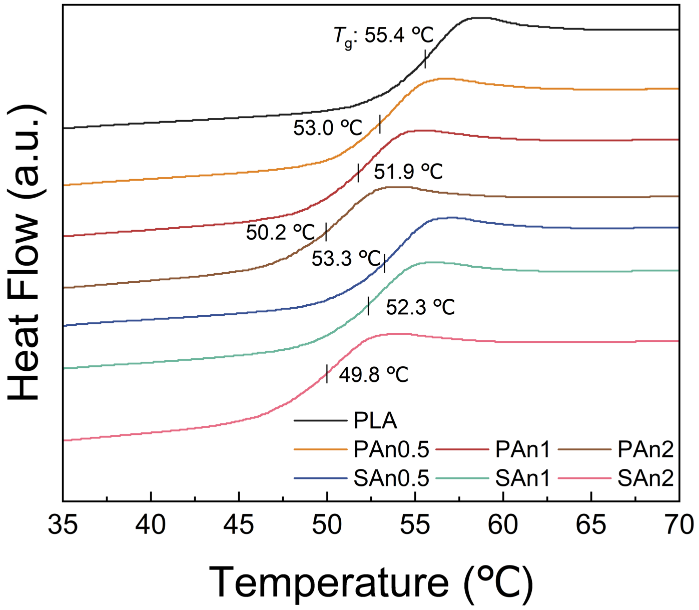

Figure S6. DSC curves (first heating, 10 °C min^{-1}) of as-prepared PLA, PAn#, and SAn#, with indicated T_g.

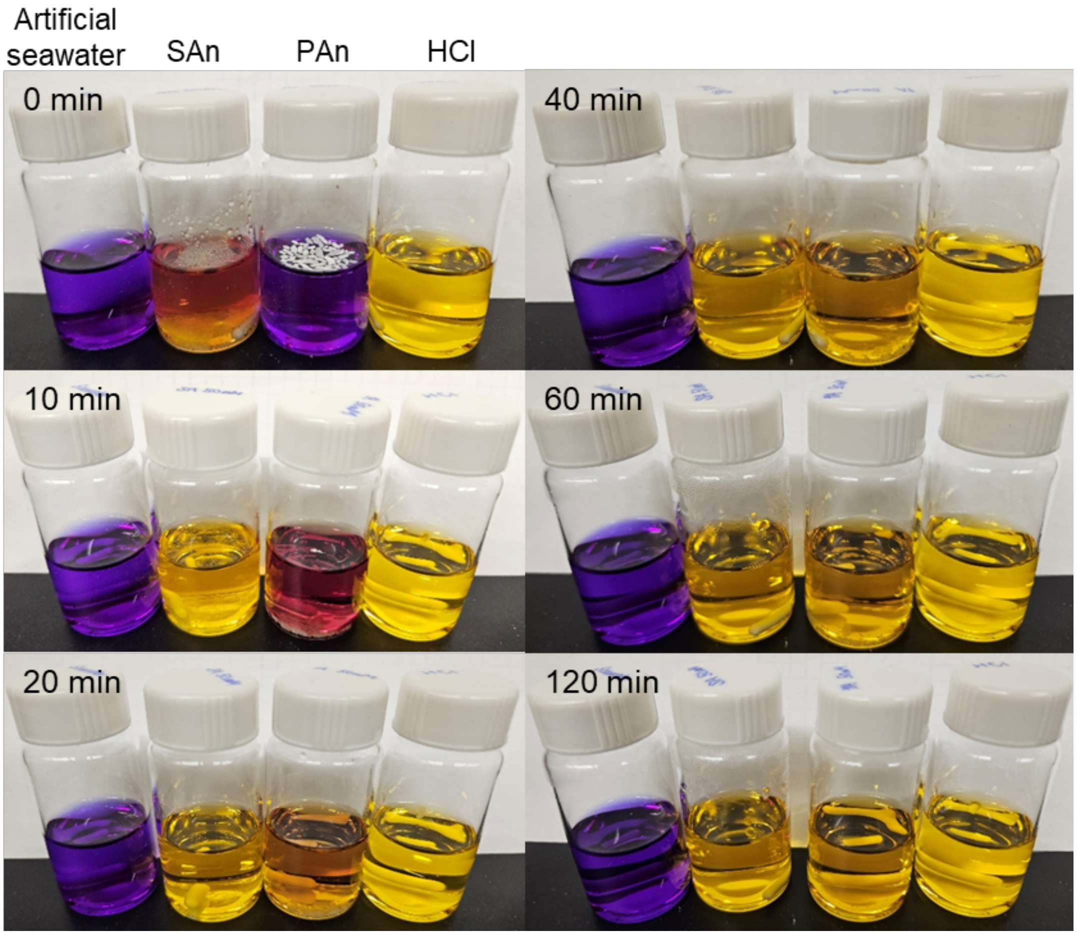

Figure S7. Time-dependent hydrolysis of PAn and SAn (50 mM) in artificial seawater with bromophenol blue (0.1 mM) as a pH indicator. Artificial seawater (Left) and a 50 mM HCl in artificial seawater (Right) were used as basic and acidic controls. SAn fully hydrolyzed by 40 min, whereas minor PAn still remained at 120 min.

Table S5. Water uptake and acid ratio of PAn and SAn stored at 20 °C under 60% relative humidity for 14 h.

Samples	Absorbed water/sample (mg/mg)	Acid ratio[a] (%)
PAn	0.0027 ± 0.0048	39.8
SAn	0.64 ± 0.29	95.0

[a]Acid ratio was determined from ^{1}H NMR analysis.

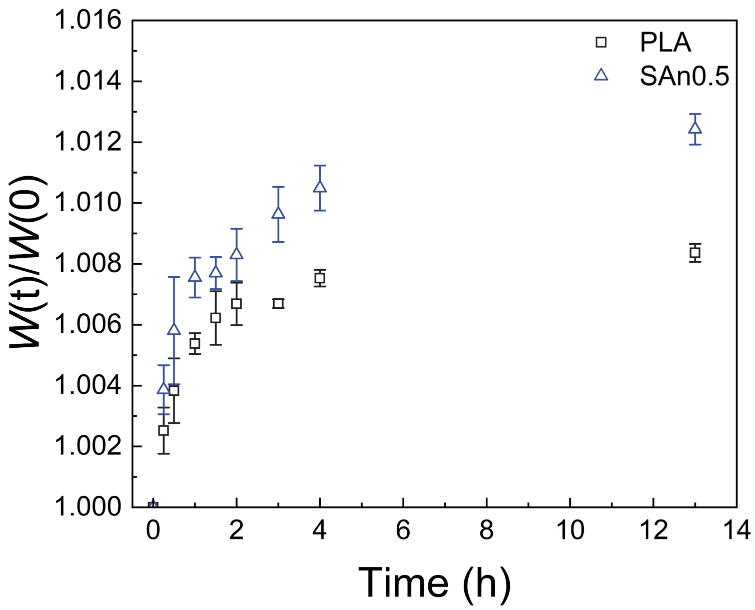

Figure S8. Fractional mass change of PLA and SAn0.5 in distilled water at the ambient temperature. The water diffusion coefficient was calculated based on the initial mass increase using the simplified Fickian diffusion model:

$$\frac{W(t) - W(0)}{W_{eq} - W(0)} = 4\sqrt{\frac{Dt}{\pi L^2}}$$

where D is the diffusion coefficient, L is the half-thickness of the films (0.165 mm), and $W(t)$, $W(0)$, and W_{eq} correspond to the mass at time t, initial mass, and equilibrium mass, respectively. [7]

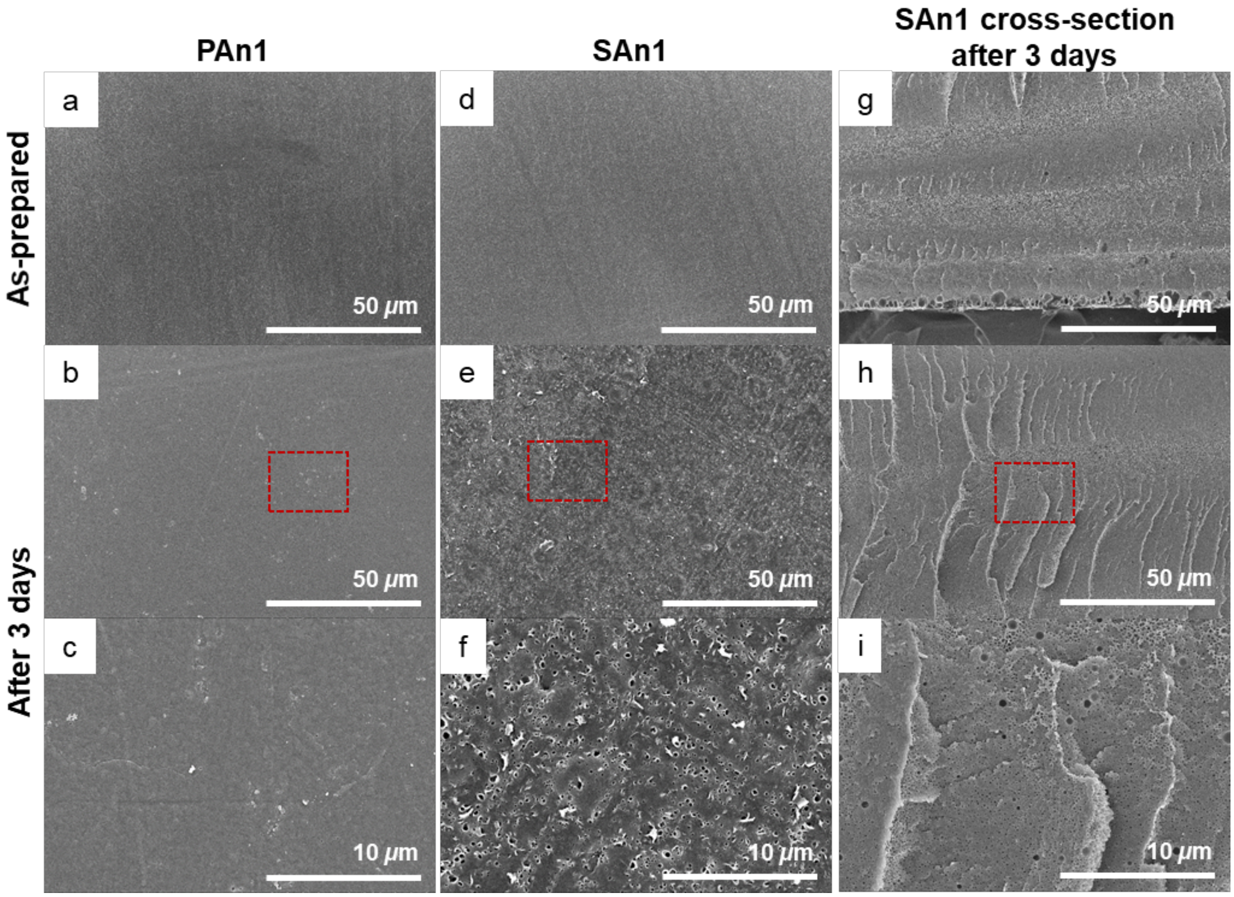

Figure S9. Surface SEM images of (a) as-prepared PAn1, (b) PAn1 immersed in artificial seawater at 50 °C for 3 days, (d) as-prepared SAn1, and (e) SAn1 immersed in artificial seawater at 50 °C for 3 days. (c) and (f) are magnified images of (b) and (e), respectively. Cross-sectional SEM images of SAn1 immersed in artificial seawater at 50 °C for 3 days (g) near the surface and (h) middle. (i) is magnified view of (h).

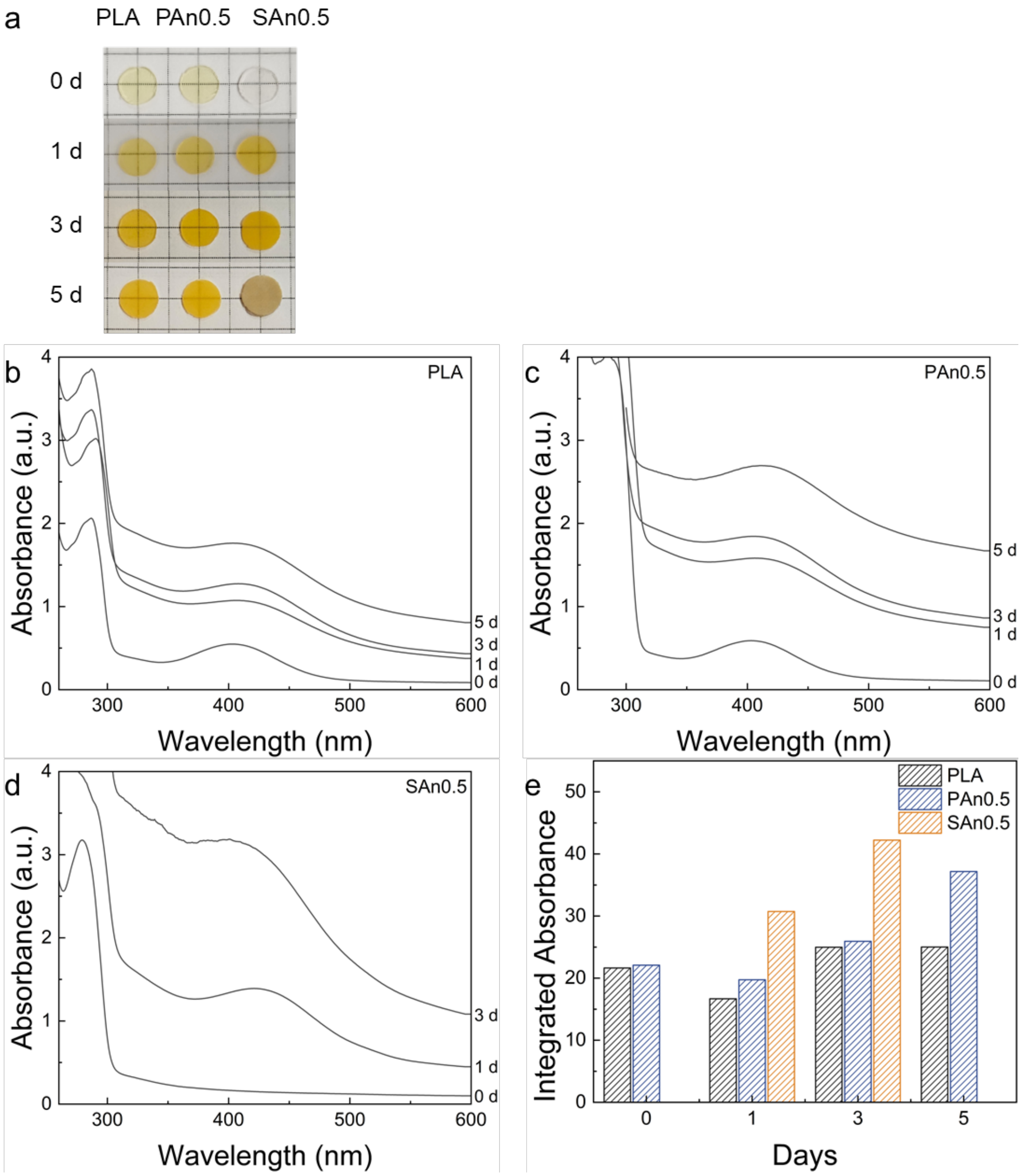

Figure S10. (a) Photographic images of PLA, PAn0.5, and SAn0.5 containing 0.5 wt% bromophenol blue as a pH indicator during hydrolytic degradation in artificial seawater at 50 °C. Representative absorption spectra of (b) PLA, (c) PAn0.5, and (d) SAn0.5 during hydrolytic degradation. The number on the right of each curve indicates the immersion time in artificial seawater at 50 °C. The overall absorbance in all samples increased over time, likely due to the water uptake, which can lead to increased light scattering and changes in effective optical path length. (e) Integrated absorbance of the band between 350 and 500 nm as a function of time.

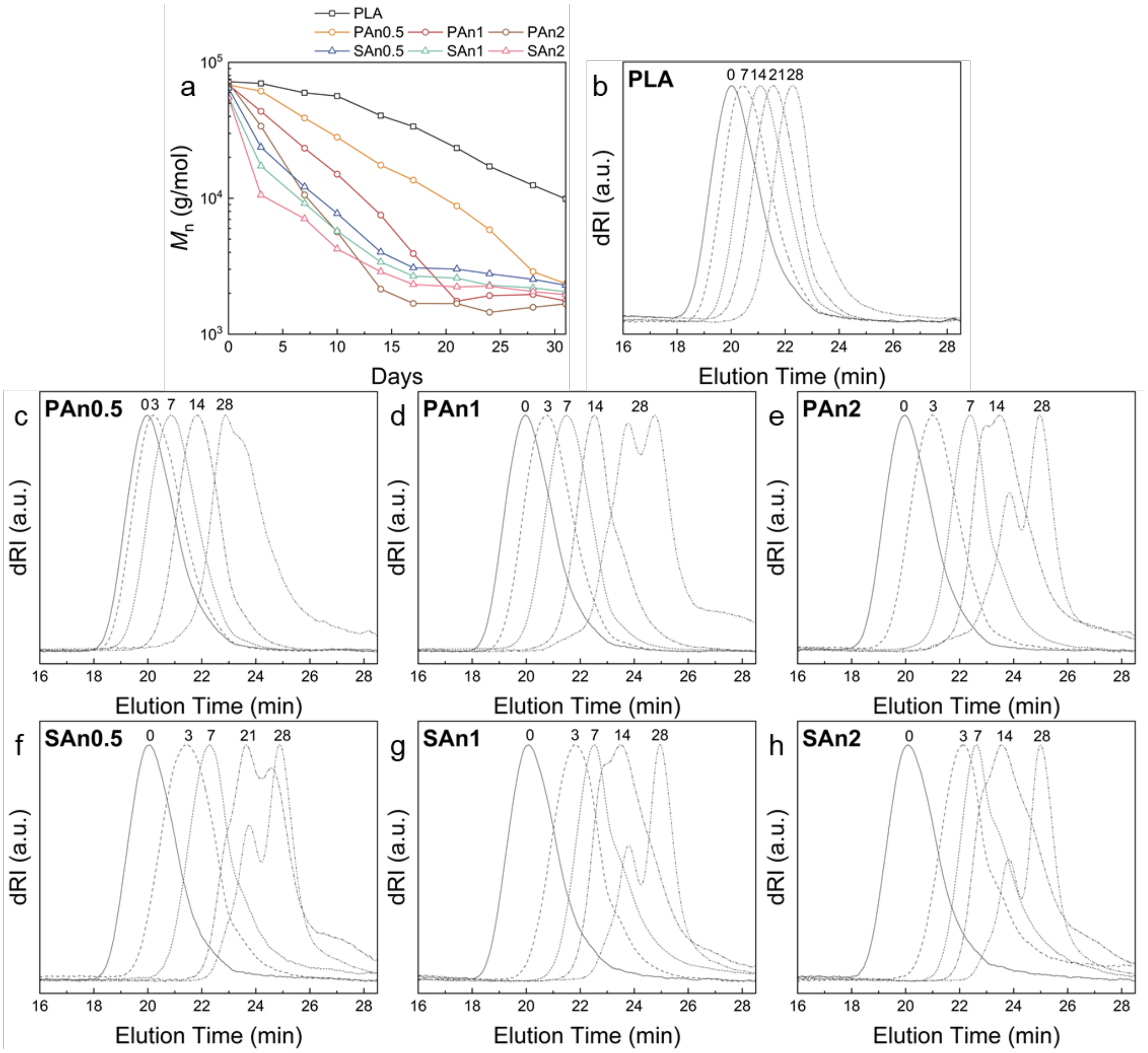

Figure S11. (a) M_n of PLA, PAn#, and SAn# as a function of hydrolysis time determined by SEC. SEC traces of (b) PLA, (c) PAn0.5, (d) PAn1, (e) PAn2, (f) SAn0.5, (g) SAn1, and (h) SAn2. Numbers above each peak represent hydrolysis time (days) at 50 °C in artificial seawater.

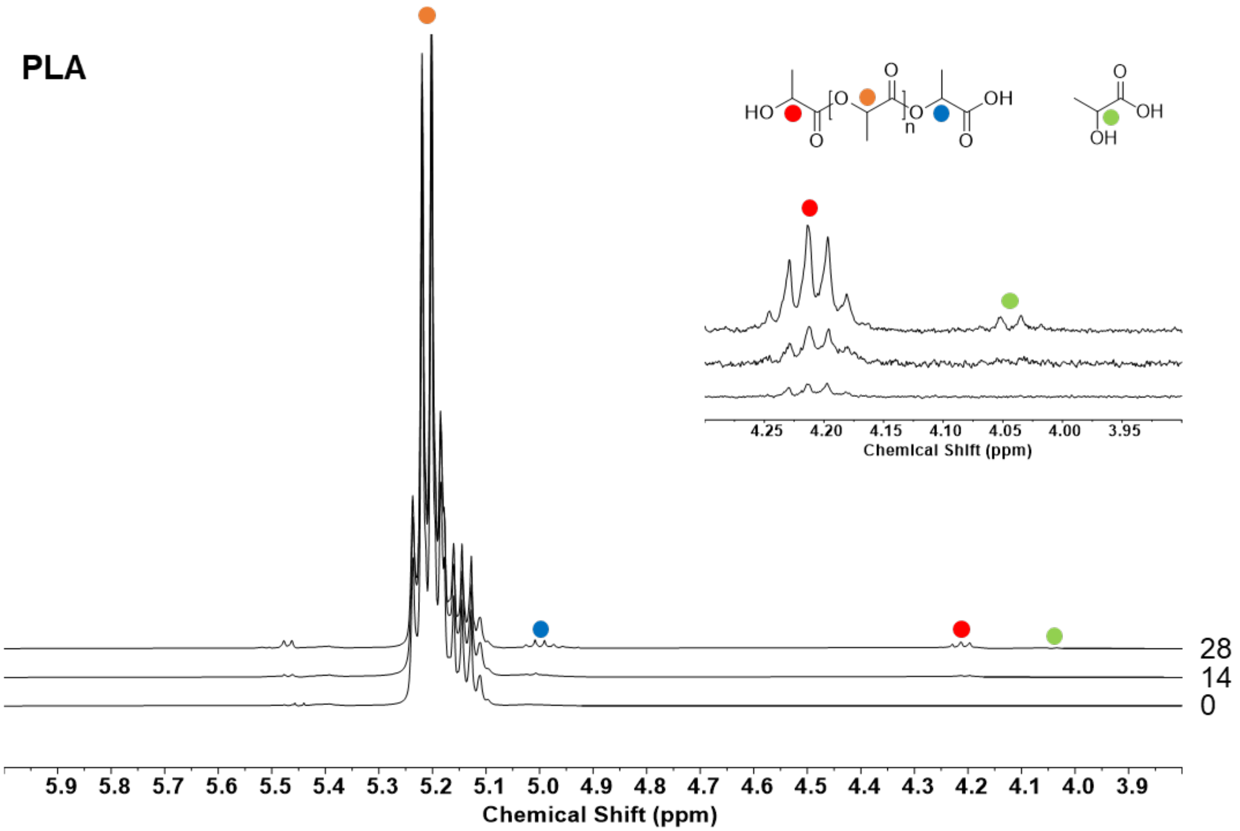

Figure S12. ^{1}H NMR spectra of PLA in the range of 3.8–6.0 ppm normalized to the polylactide repeating unit methine signal (5.1–5.3 ppm) (recorded in DMSO-d_6). Numbers on the right indicate the hydrolysis time (days) at 50 °C in artificial seawater. The inset highlights the region of 3.9–4.3 ppm, illustrating the generation of lactic acid.

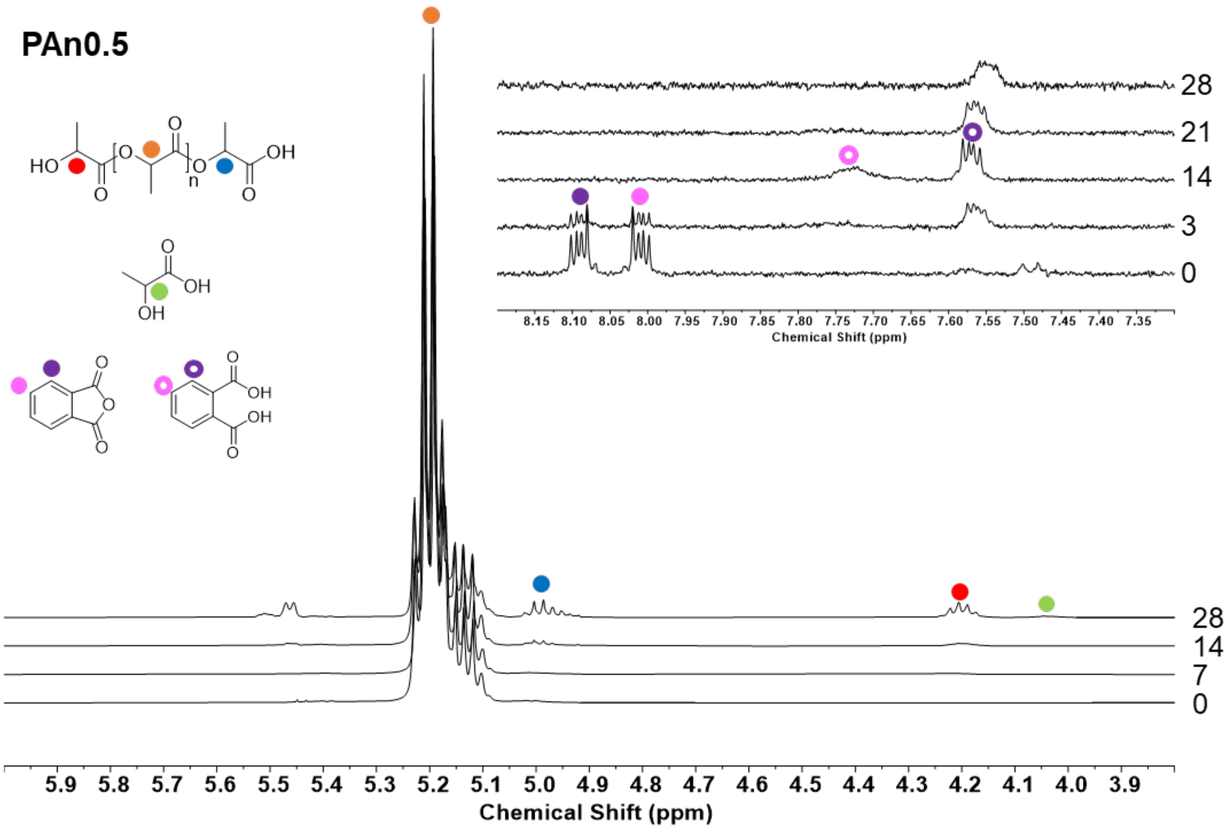

Figure S13. ^{1}H NMR spectra of PAn0.5 in the range of 3.8–6.0 ppm recorded in DMSO-d_6. Numbers on the right indicate the hydrolysis time (days) at 50 °C in artificial seawater. The inset displays the aromatic region (7.3–8.2 ppm), showing the spectral changes associated with the additive during hydrolysis.

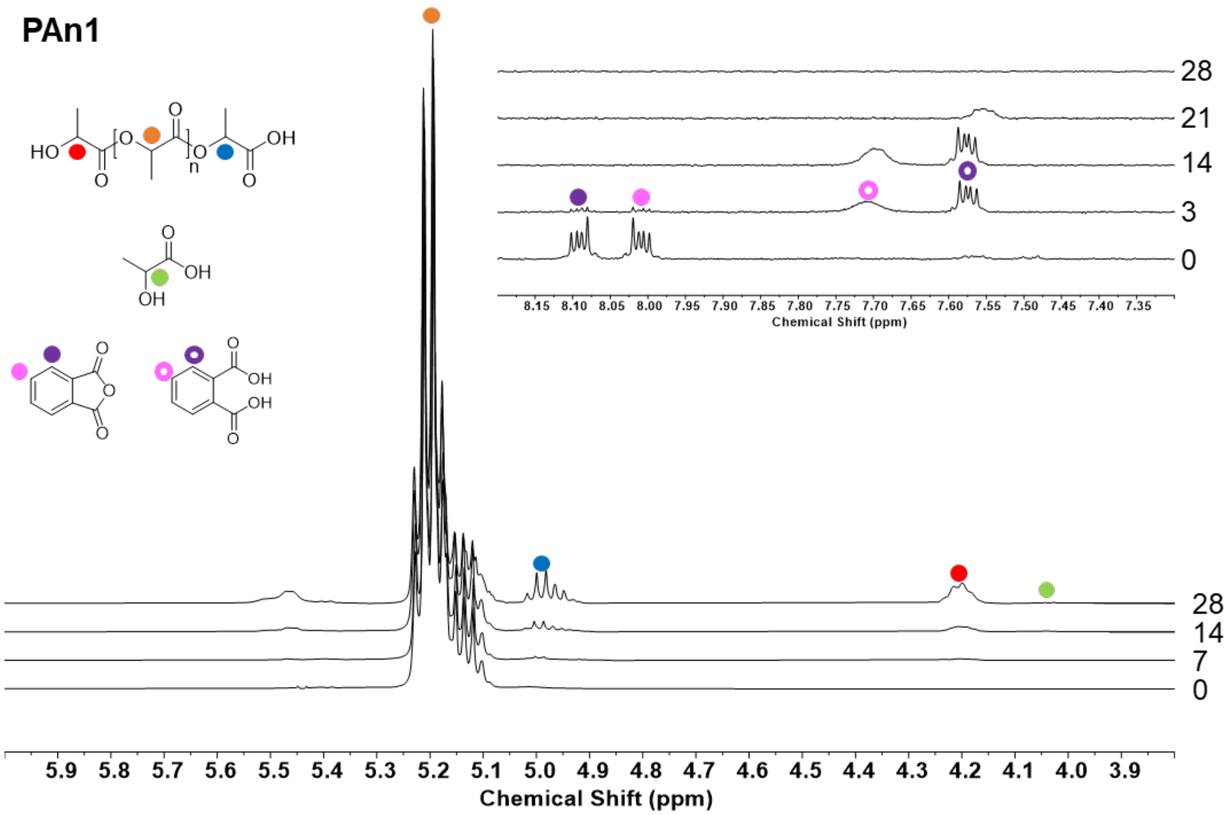

Figure S14. ^{1}H NMR spectra of PAn1 in the range of 3.8–6.0 ppm recorded in DMSO-d_6. Numbers on the right indicate the hydrolysis time (days) at 50 °C in artificial seawater. The inset displays the aromatic region (7.3–8.2 ppm), showing the spectral changes associated with the additive during hydrolysis.

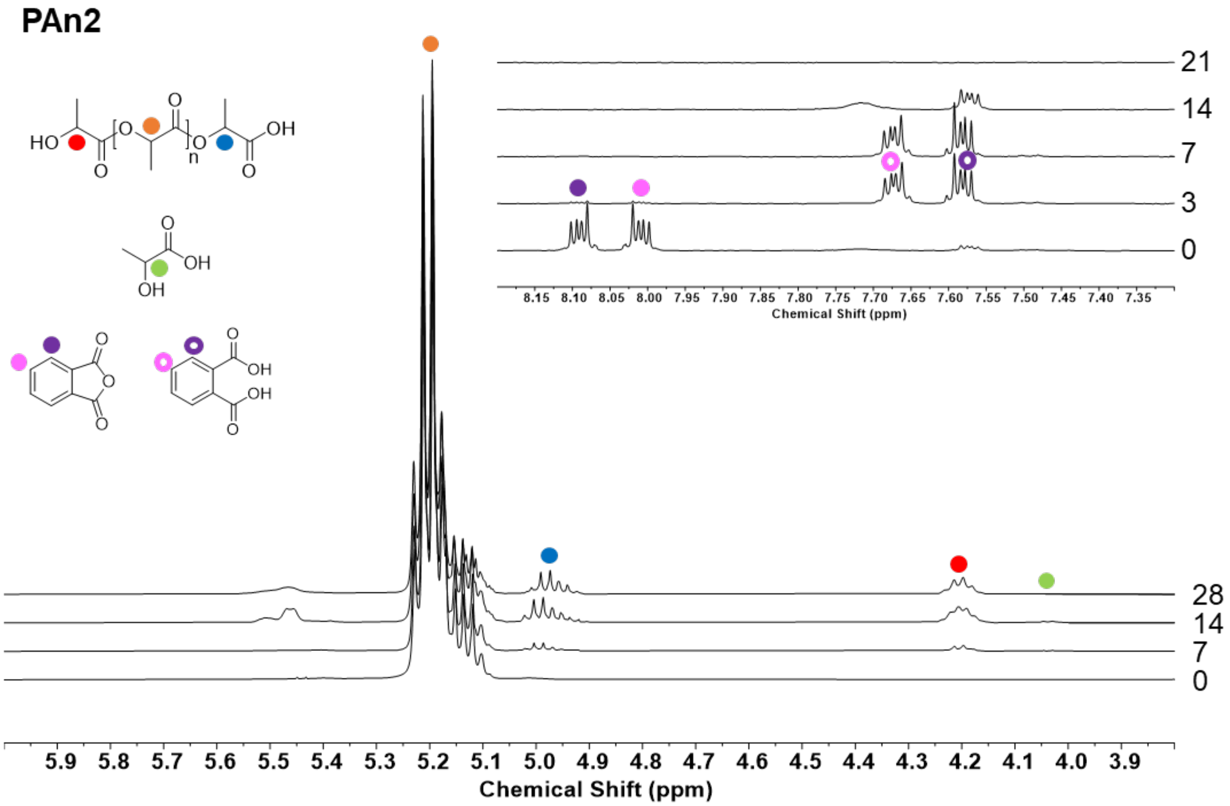

Figure S15. ^{1}H NMR spectra of PAn2 in the range of 3.8–6.0 ppm recorded in DMSO-d_6. Numbers on the right indicate the hydrolysis time (days) at 50 °C in artificial seawater. The inset displays the aromatic region (7.3–8.2 ppm), showing the spectral changes associated with the additive during hydrolysis.

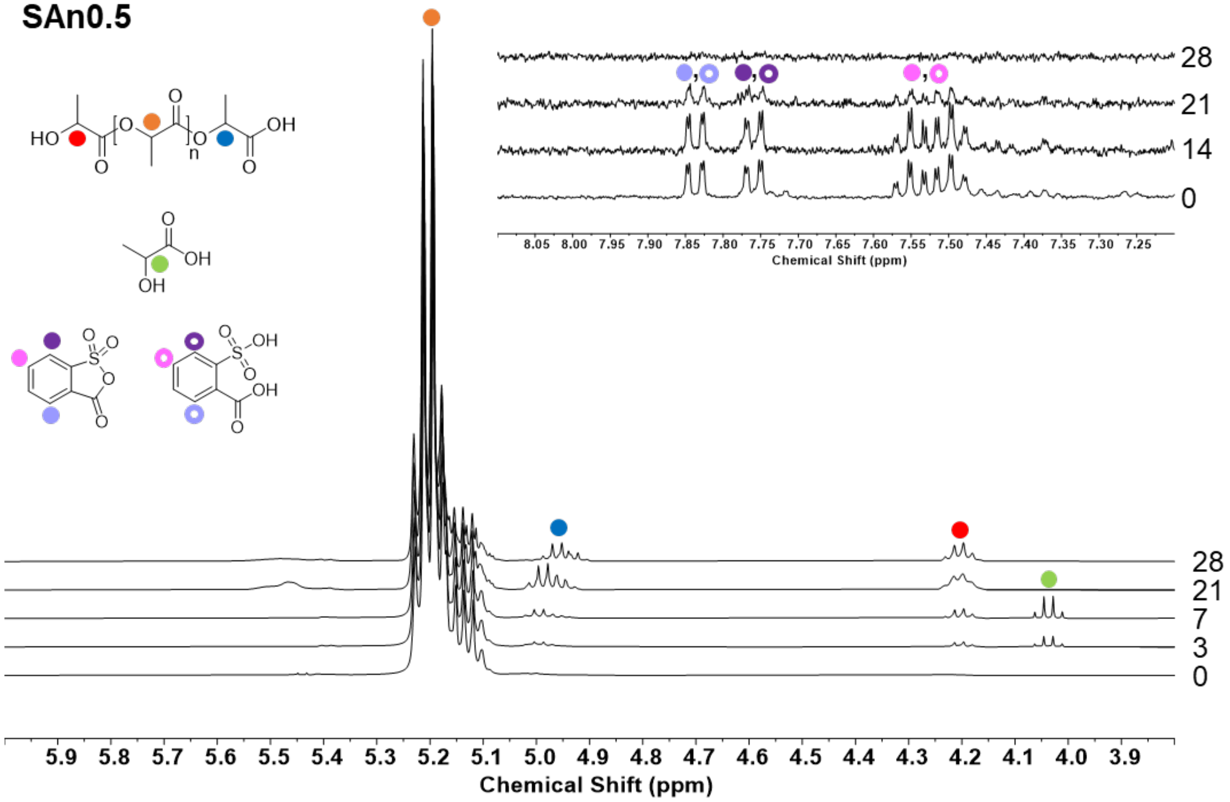

Figure S16. ^{1}H NMR spectra of SAn0.5 in the range of 3.8–6.0 ppm recorded in DMSO-d_6. Numbers on the right indicate the hydrolysis time (days) at 50 °C in artificial seawater. The inset displays the aromatic region (7.2–8.1 ppm), showing the spectral changes associated with the additive during hydrolysis.

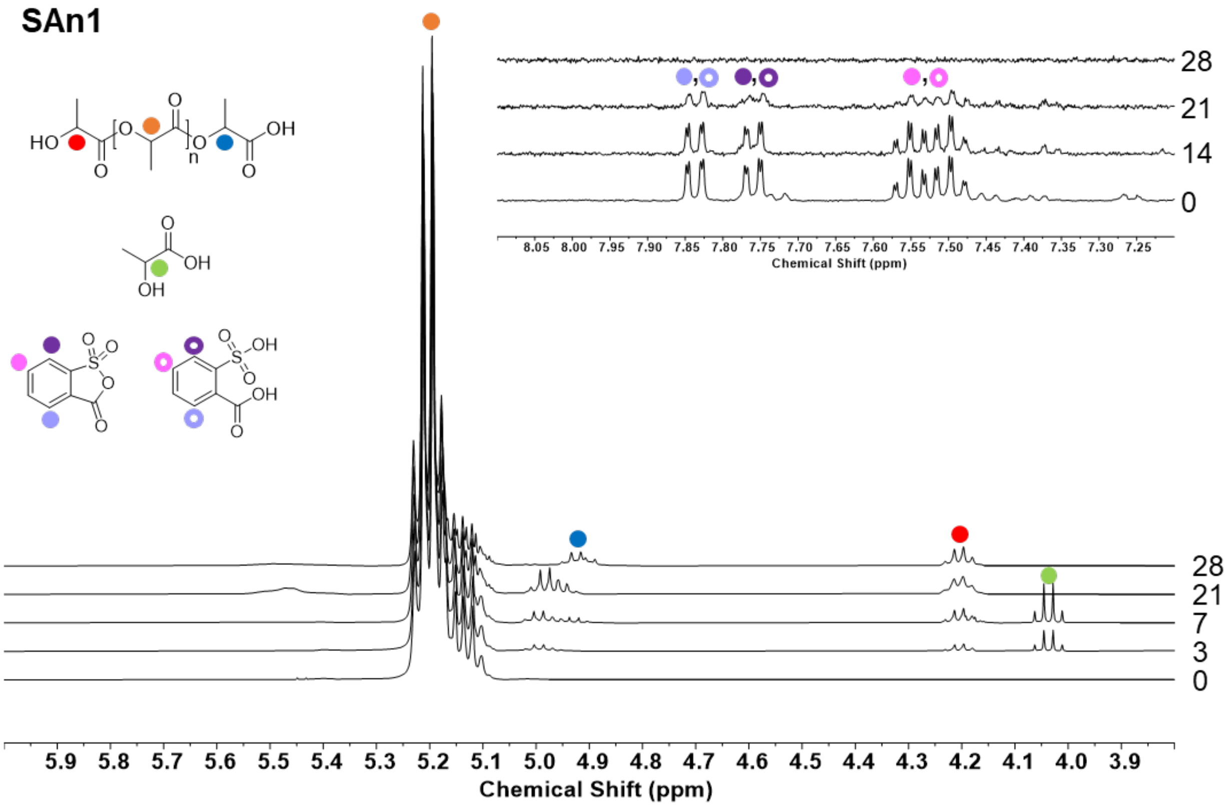

Figure S17. ^{1}H NMR spectra of SAn1 in the range of 3.8–6.0 ppm recorded in DMSO-d_6. Numbers on the right indicate the hydrolysis time (days) at 50 °C in artificial seawater. The inset displays the aromatic region (7.2–8.1 ppm), showing the spectral changes associated with the additive during hydrolysis.

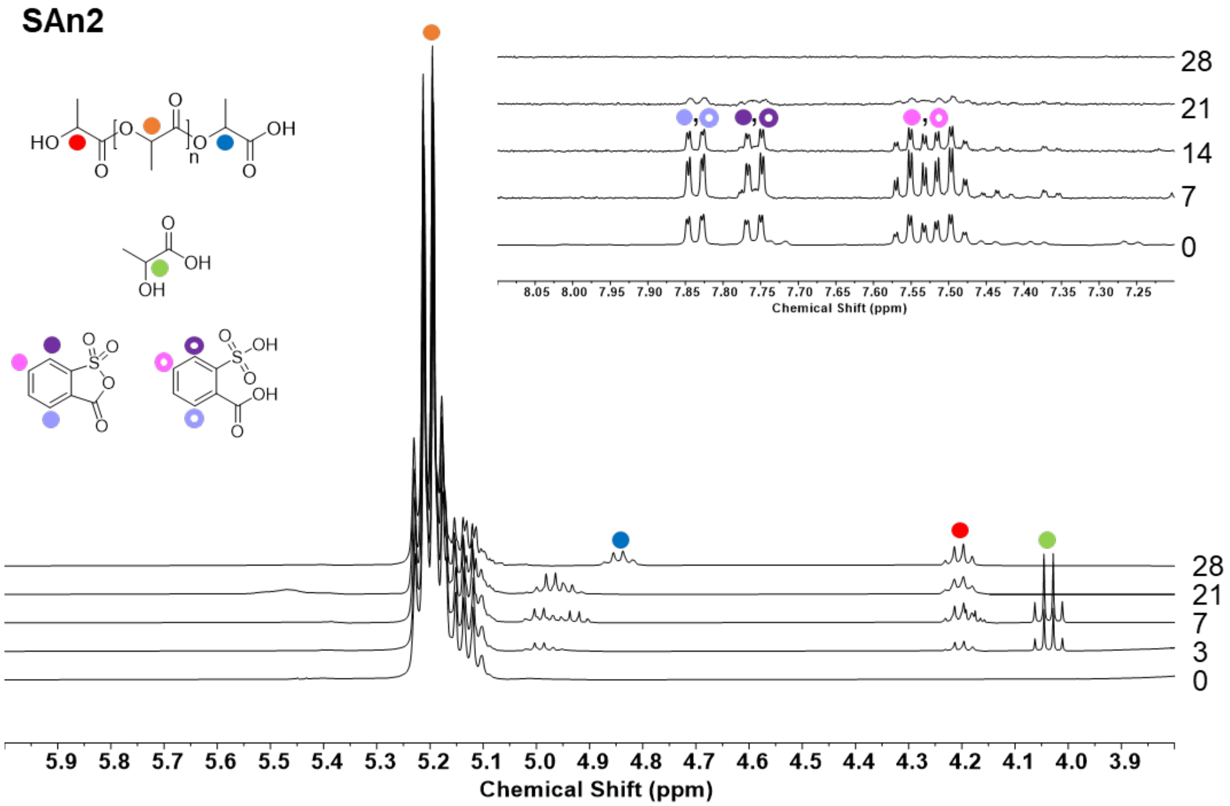

Figure S18. ^{1}H NMR spectra of SAn2 in the range of 3.8–6.0 ppm recorded in DMSO-d_6. Numbers on the right indicate the hydrolysis time (days) at 50 °C in artificial seawater. The inset displays the aromatic region (7.2–8.1 ppm), showing the spectral changes associated with the additive during hydrolysis.

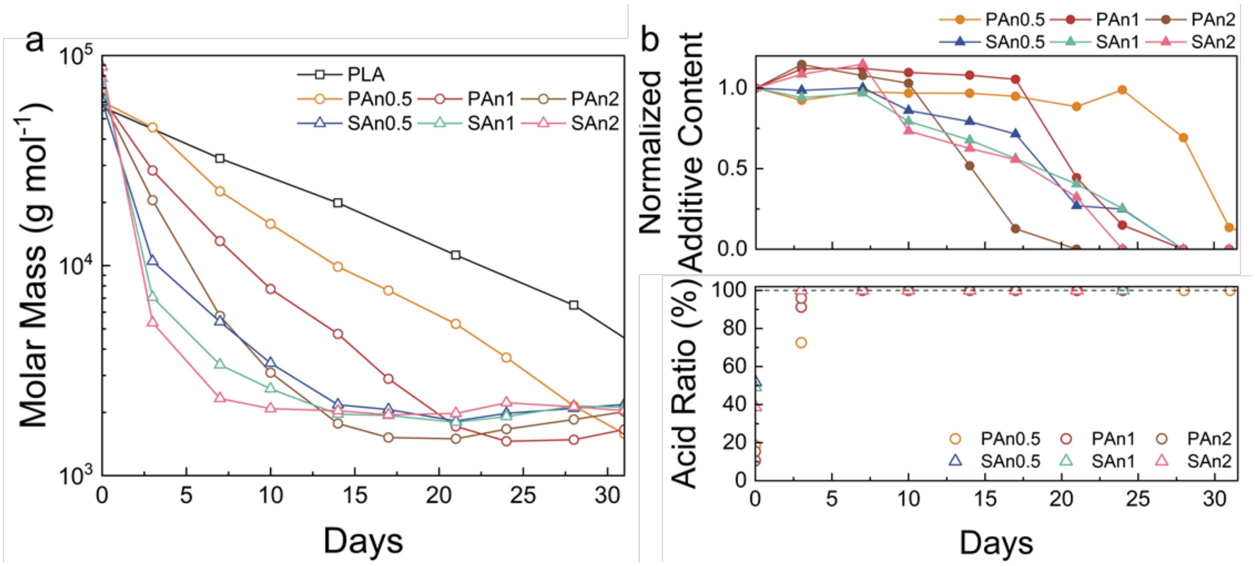

Figure S19. (a) Molar mass of PLA, PAn#, and SAn# determined by end-group analysis using ^{1}H NMR data as a function of hydrolysis time at 50 °C in artificial seawater. (b) Normalized additive content (top) and acid ratio (bottom) of PAn# and SAn# during hydrolysis.

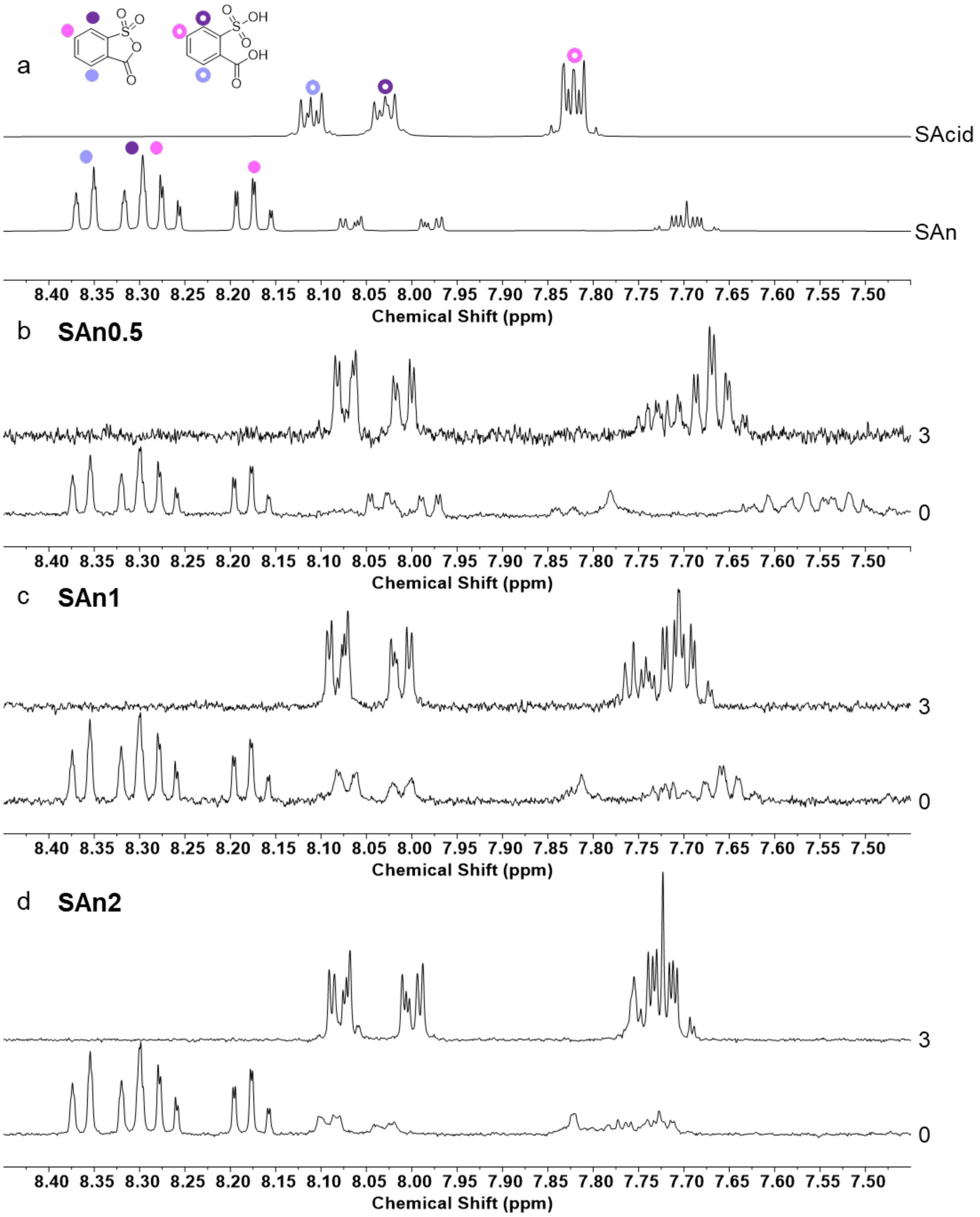

Figure S20. (a) ^{1}H NMR spectra of SAn (bottom) and SAcid (top) in the aromatic region (7.45–8.45 ppm) recorded in acetone-d_6. ^{1}H NMR spectra (b) SAn0.5, (c) SAn1, and (d) SAn2 before (bottom) and after 3 days (top) of hydrolysis at 50 °C in artificial seawater.

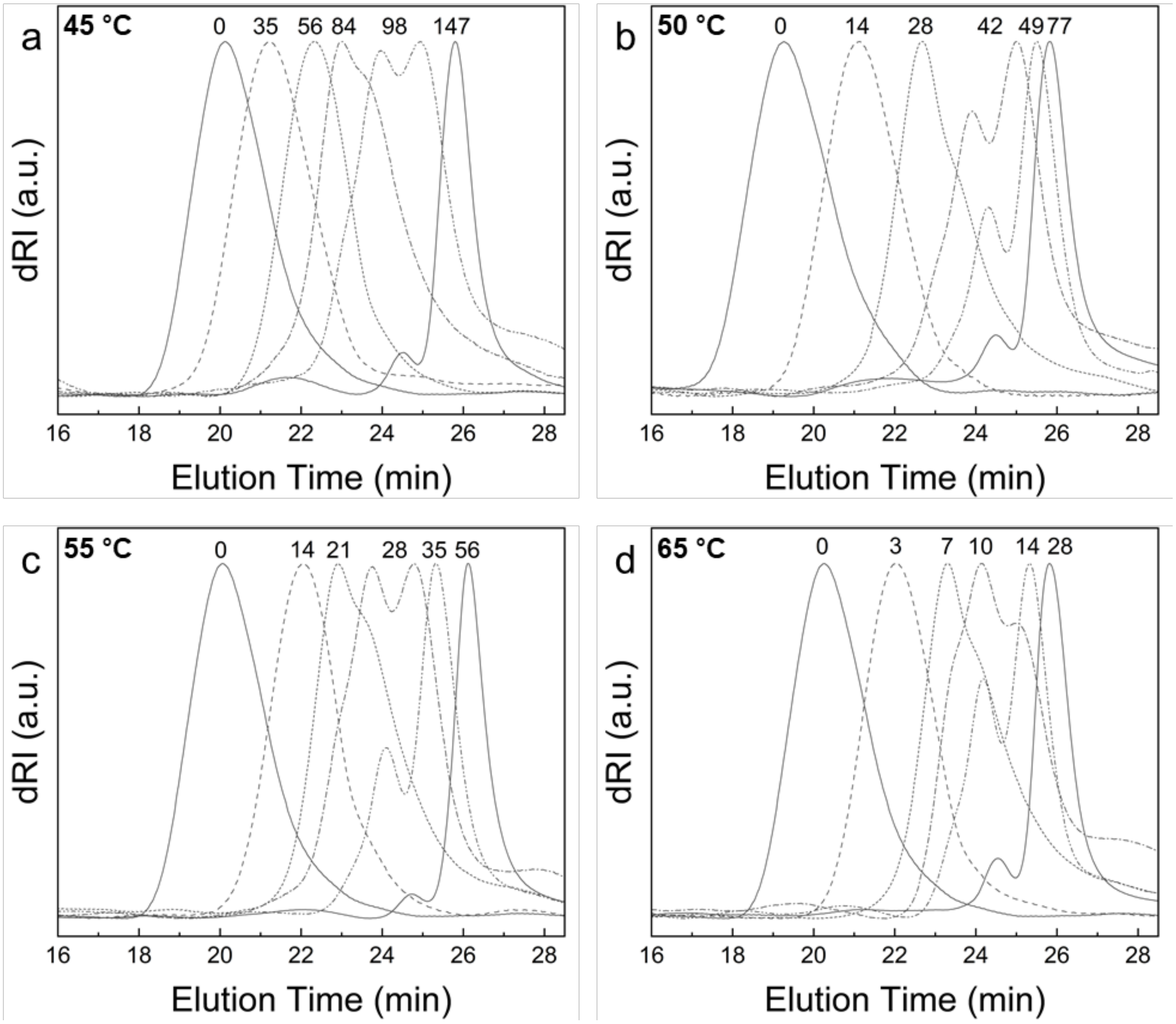

Figure S21. SEC traces of the insoluble fraction of PLA during hydrolytic degradation at (a) 45 °C, (b) 50 °C, (c) 55 °C, and (d) 65 °C. Numbers above each peak indicate the corresponding hydrolysis time (days).

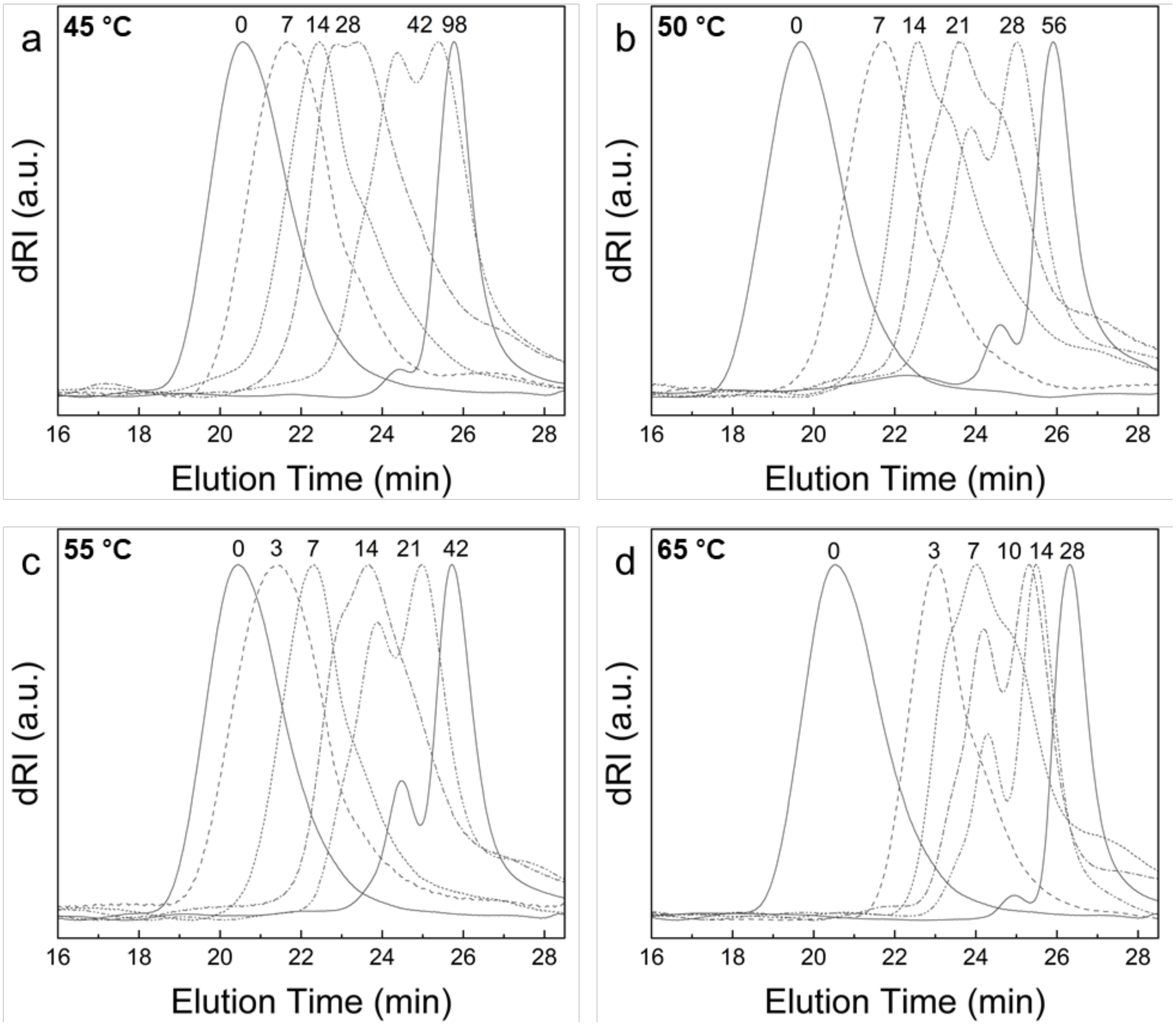

Figure S22. SEC traces of the insoluble fraction of SAn0.5 during hydrolytic degradation at (a) 45 °C, (b) 50 °C, (c) 55 °C, and (d) 65 °C. Numbers above each peak indicate the corresponding hydrolysis time (days).

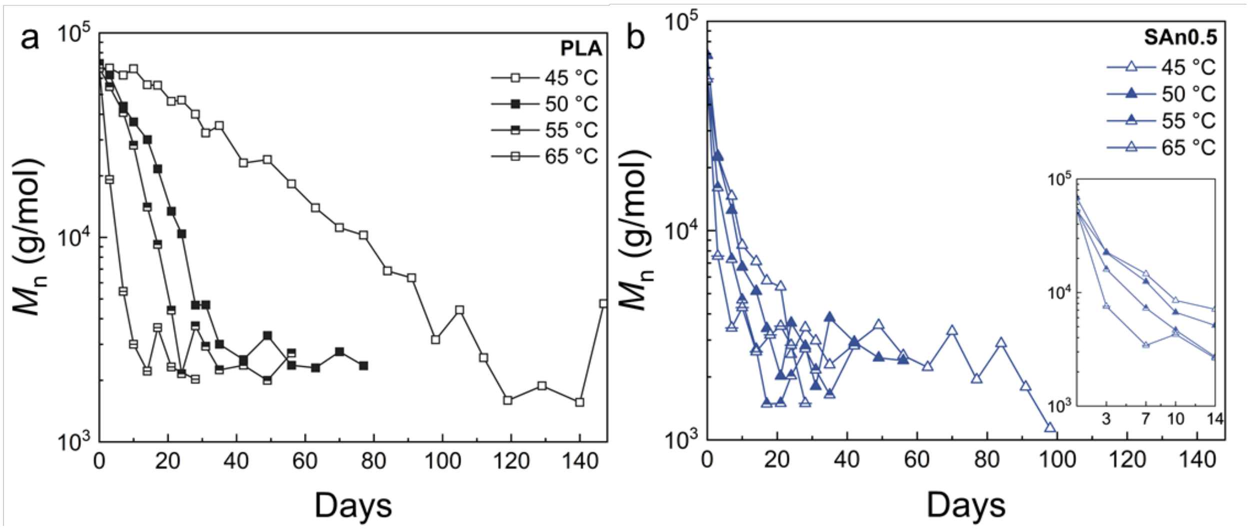

Figure S23. M_n of (a) PLA and (b) SAn0.5 as a function of hydrolysis time at various temperatures (45, 50, 55, and 65 °C) in artificial seawater, determined from SEC results. The inset in (b) highlights the M_n change of SAn0.5 over the first 14 days.

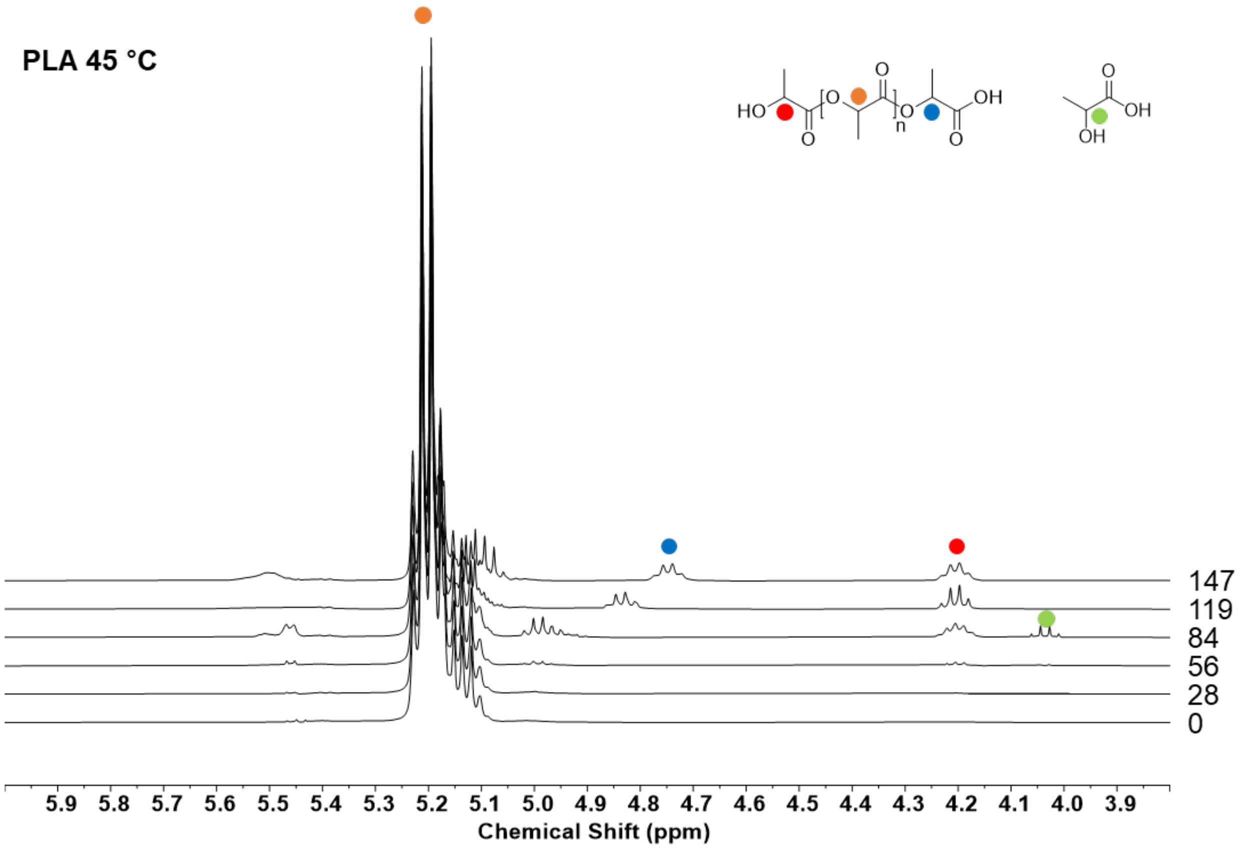

Figure S24. ^{1}H NMR spectra of PLA in the range of 3.8–6.0 ppm recorded in DMSO-d_6. Numbers on the right indicate the hydrolysis time (days) at 45 °C in artificial seawater.

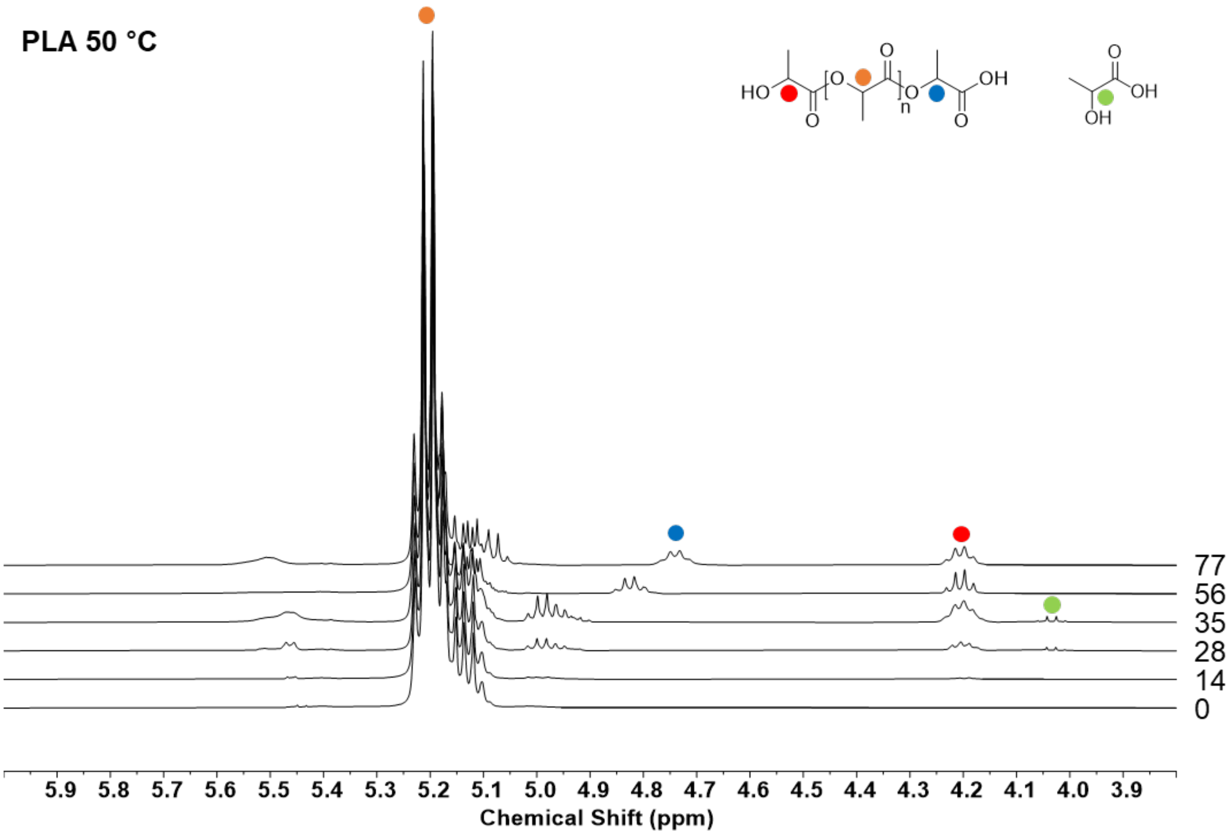

Figure S25. ^{1}H NMR spectra of PLA in the range of 3.8–6.0 ppm recorded in DMSO-d_6. Numbers on the right indicate the hydrolysis time (days) at 50 °C in artificial seawater.

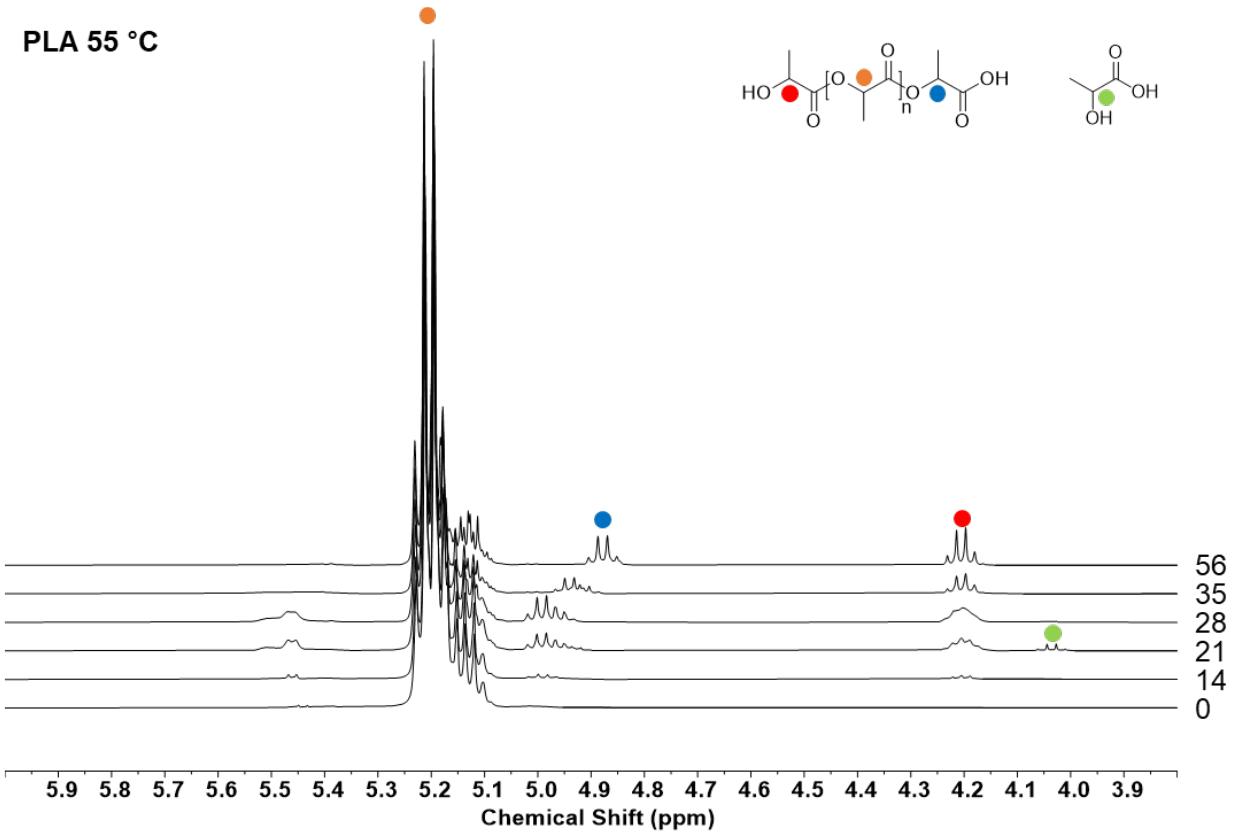

Figure S26. ^{1}H NMR spectra of PLA in the range of 3.8–6.0 ppm recorded in DMSO-d_6. Numbers on the right indicate the hydrolysis time (days) at 55 °C in artificial seawater.

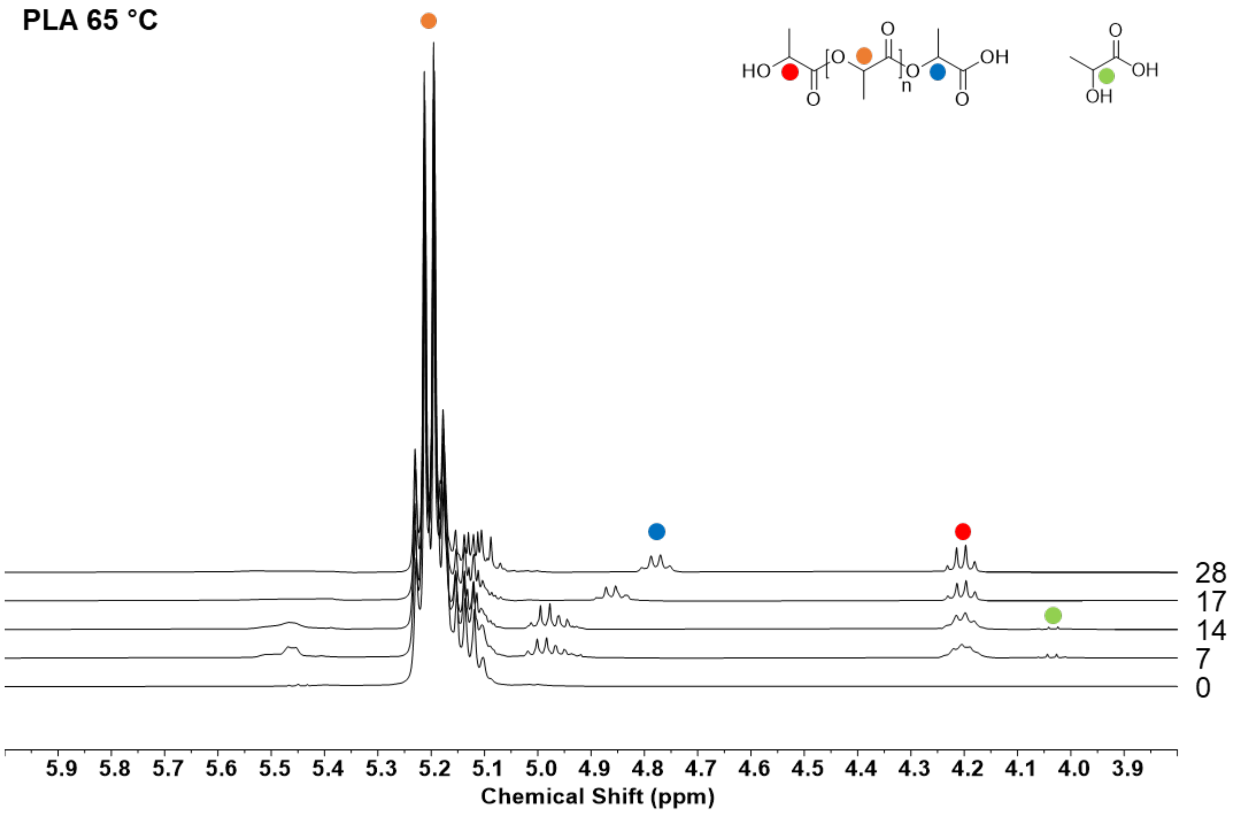

Figure S27. ^{1}H NMR spectra of PLA in the range of 3.8–6.0 ppm recorded in DMSO-d_6. Numbers on the right indicate the hydrolysis time (days) at 65 °C in artificial seawater.

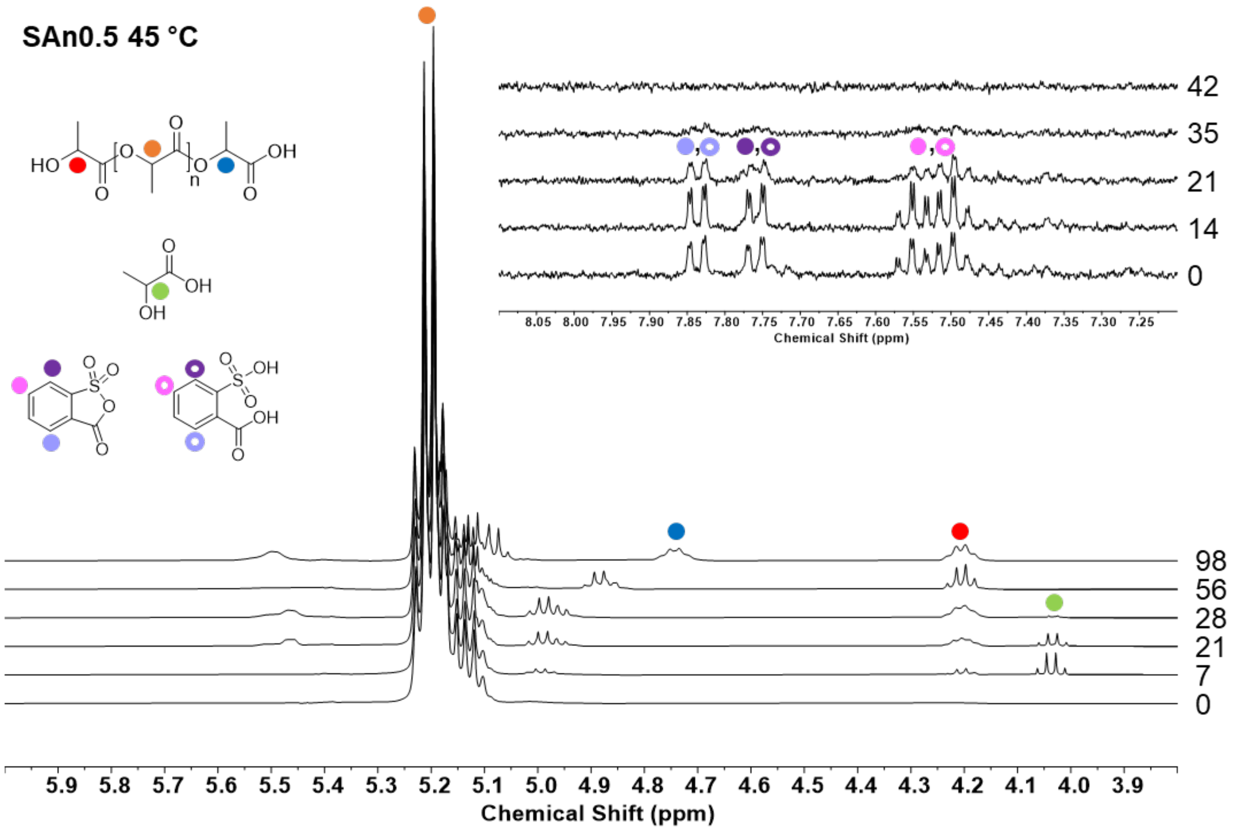

Figure S28. ^{1}H NMR spectra of SAn0.5 in the range of 3.8–6.0 ppm recorded in DMSO-d_6. Numbers on the right indicate the hydrolysis time (days) at 45 °C in artificial seawater. The inset displays the aromatic region (7.2–8.1 ppm), showing the spectral changes associated with the additive during hydrolysis.

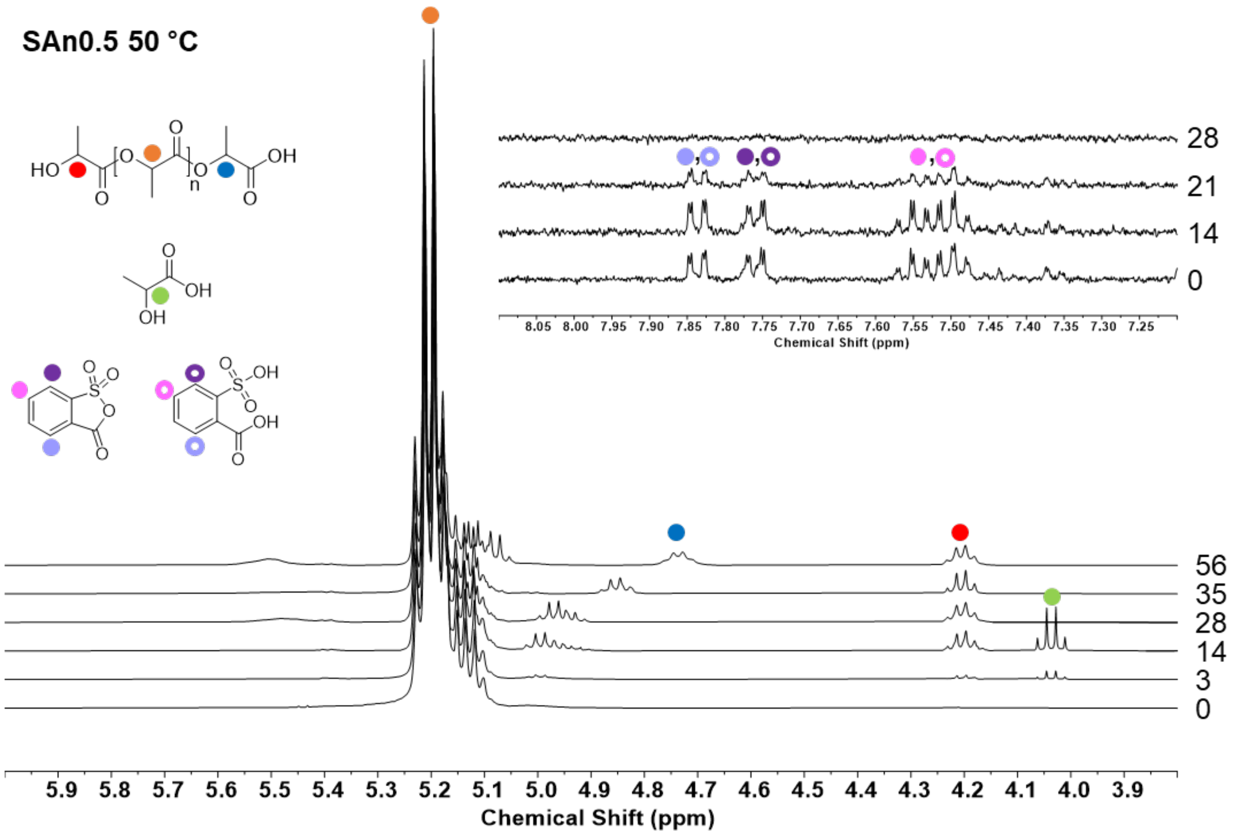

Figure S29. ^{1}H NMR spectra of SAn0.5 in the range of 3.8–6.0 ppm recorded in DMSO-d_6. Numbers on the right indicate the hydrolysis time (days) at 50 °C in artificial seawater. The inset displays the aromatic region (7.2–8.1 ppm), showing the spectral changes associated with the additive during hydrolysis.

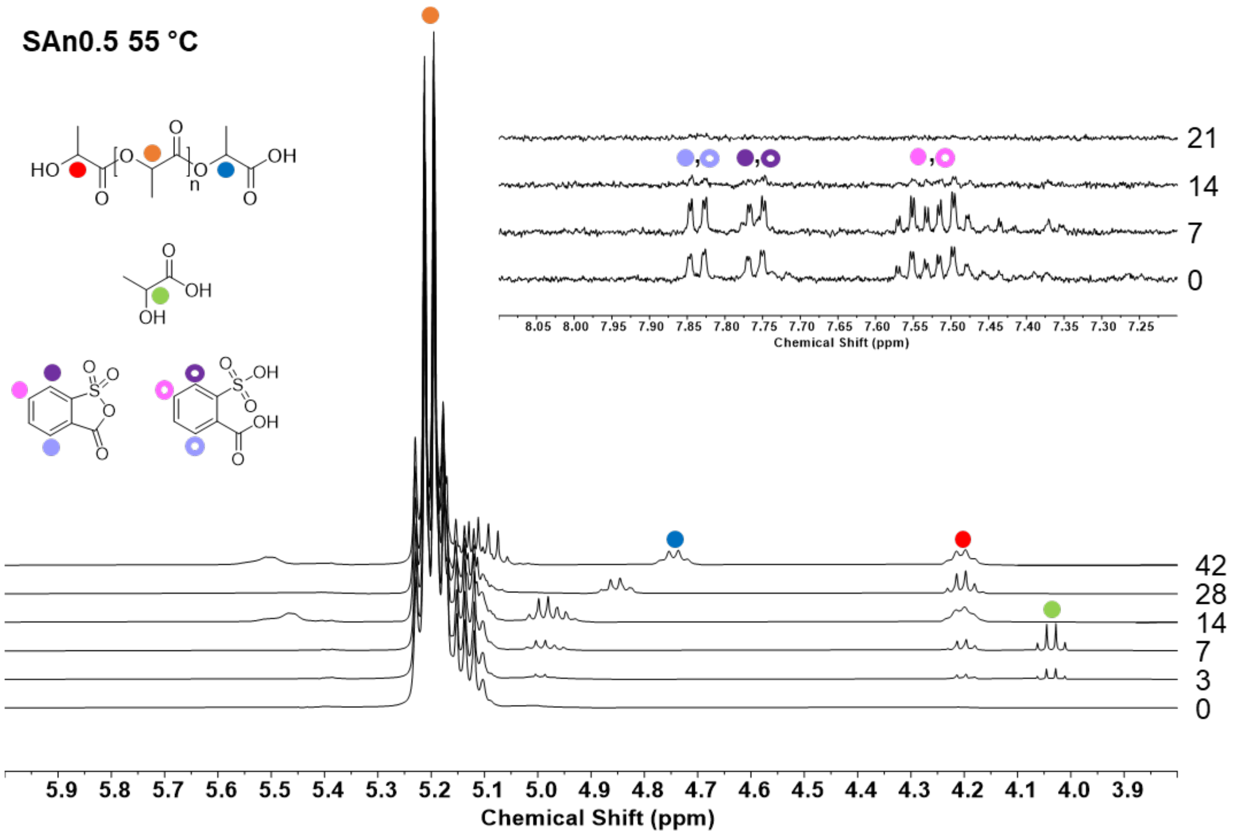

Figure S30. ^{1}H NMR spectra of SAn0.5 in the range of 3.8–6.0 ppm recorded in DMSO-d_6. Numbers on the right indicate the hydrolysis time (days) at 55 °C in artificial seawater. The inset displays the aromatic region (7.2–8.1 ppm), showing the spectral changes associated with the additive during hydrolysis.

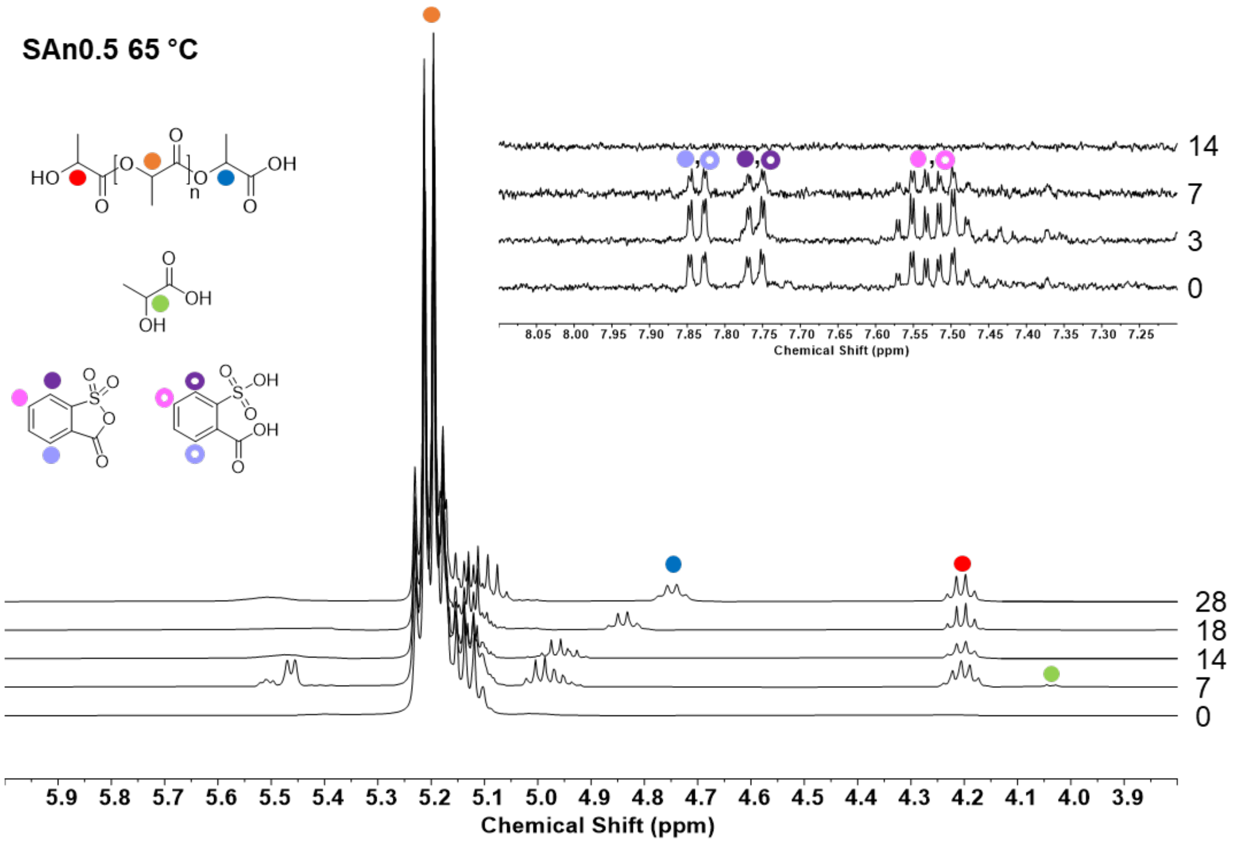

Figure S31. ^{1}H NMR spectra of SAn0.5 in the range of 3.8–6.0 ppm recorded in DMSO-d_6. Numbers on the right indicate the hydrolysis time (days) at 65 °C in artificial seawater. The inset displays the aromatic region (7.2–8.1 ppm), showing the spectral changes associated with the additive during hydrolysis.

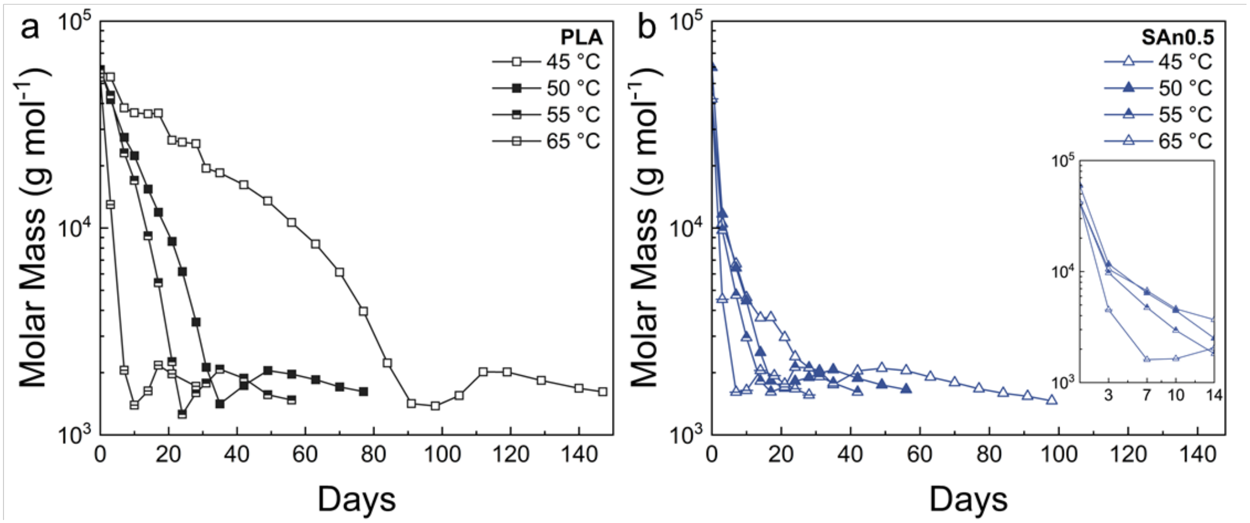

Figure S32. Molar mass of (a) PLA and (b) SAn0.5 as a function of hydrolysis time at different temperatures (45, 50, 55, and 65 °C) in artificial seawater, determined by end-group analysis using ^{1}H NMR data. The inset in (b) highlights the molar mass change of SAn0.5 during the first 14 days. The slight increase in molar mass after reaching the minimum value can be attributed to the elution of water-soluble small molecules or oligomers that had accumulated within the solid matrix until a critical molar mass was reached.

Table S6. Hydrolysis rate constants of PLA and SAn0.5 at 50 °C.

Samples	k (day^{-1})
PLA	0.10
SAn0.5	0.54/ 0.19[a]

[a] k_1/k_2

The rate constants were calculated using the following equation:

$$M_n(t) = M_n(0)e^{-kt}$$

where k is the rate constant, t is time, and $M_n(t)$ and $M_n(0)$ represent molar mass determined from ^{1}H NMR at time t and at the initial state, respectively. [8]

For SAn0.5, k_1 corresponds to the initial three-day period and k_2 to the subsequent degradation stage until the molar mass reached 1.5 kg mol^{-1}. We note that $M_n(3)$ was used as the reference initial value for the k_2 calculation, rather than $M_n(0)$. Two separate rate constants were employed because SAn0.5 exhibited two-stage degradation behavior, characterized by an initial rapid decrease in molar mass, followed by a slightly slower molar mass reduction. This behavior is attributed to pronounced water uptake and accompanying volume expansion at short times, and subsequent release of acidic degradation products into the surrounding aqueous environment.

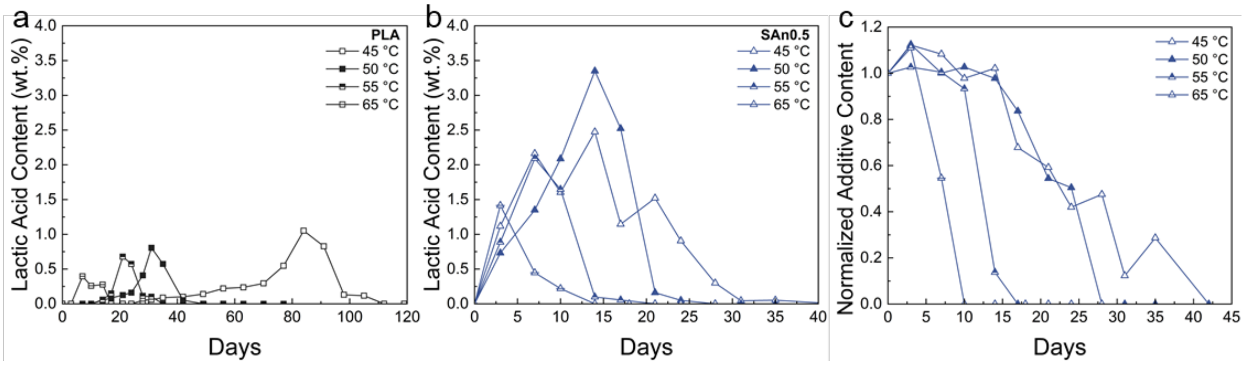

Figure S33. Content of lactic acid in (a) PLA and (b) SAn0.5 generated during hydrolytic degradation at different temperatures (45, 50, 55, and 65 °C), as determined by ^{1}H NMR analysis. (c) Normalized additive content in SAn0.5 during hydrolytic degradation at various temperatures, determined from ^{1}H NMR spectra.

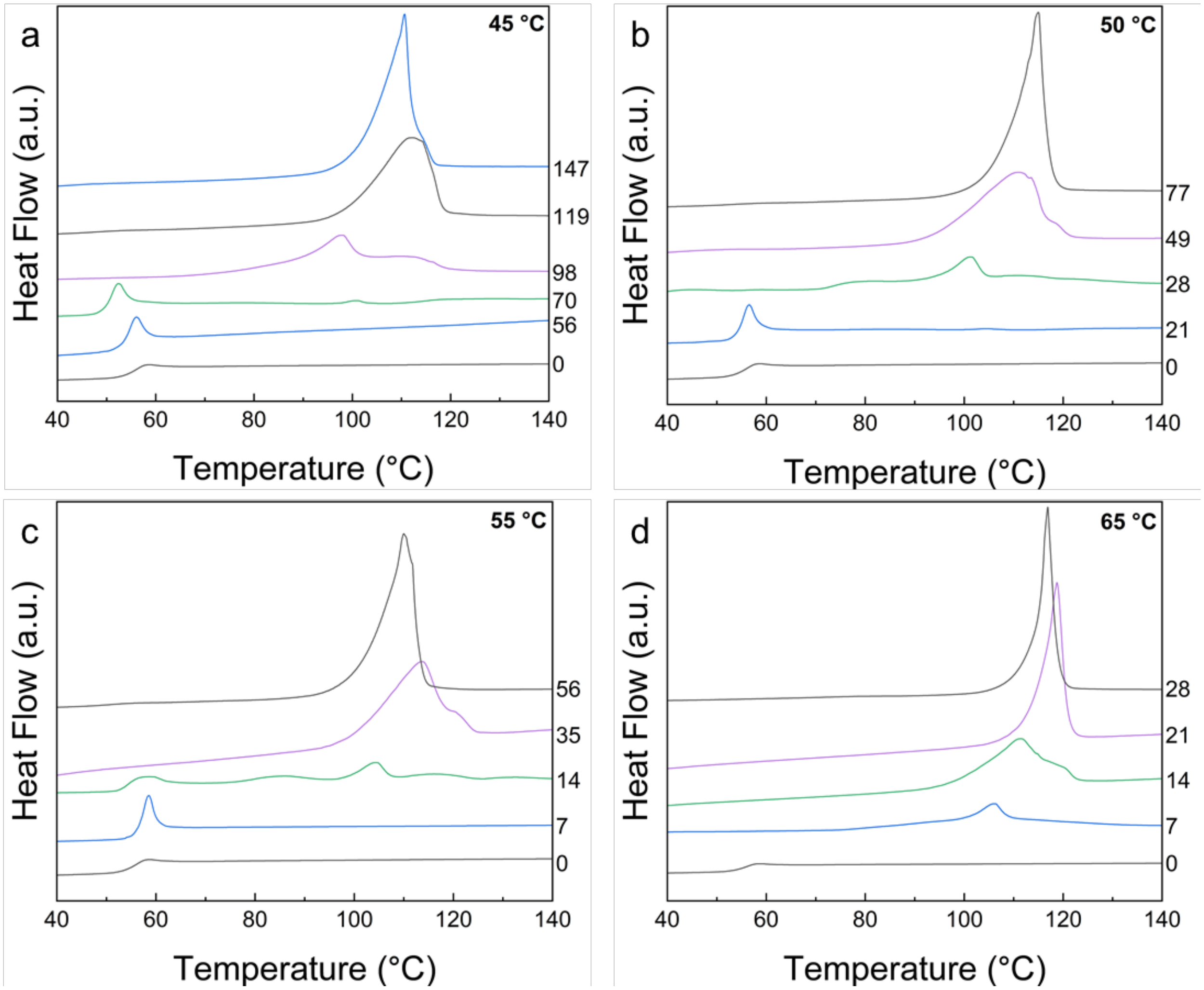

Figure S34. DSC 1st heating curves of PLA during hydrolytic degradation at (a) 45 °C, (b) 50 °C, (c) 55 °C, and (d) 65 °C. Numbers on the right indicate hydrolysis time (days).

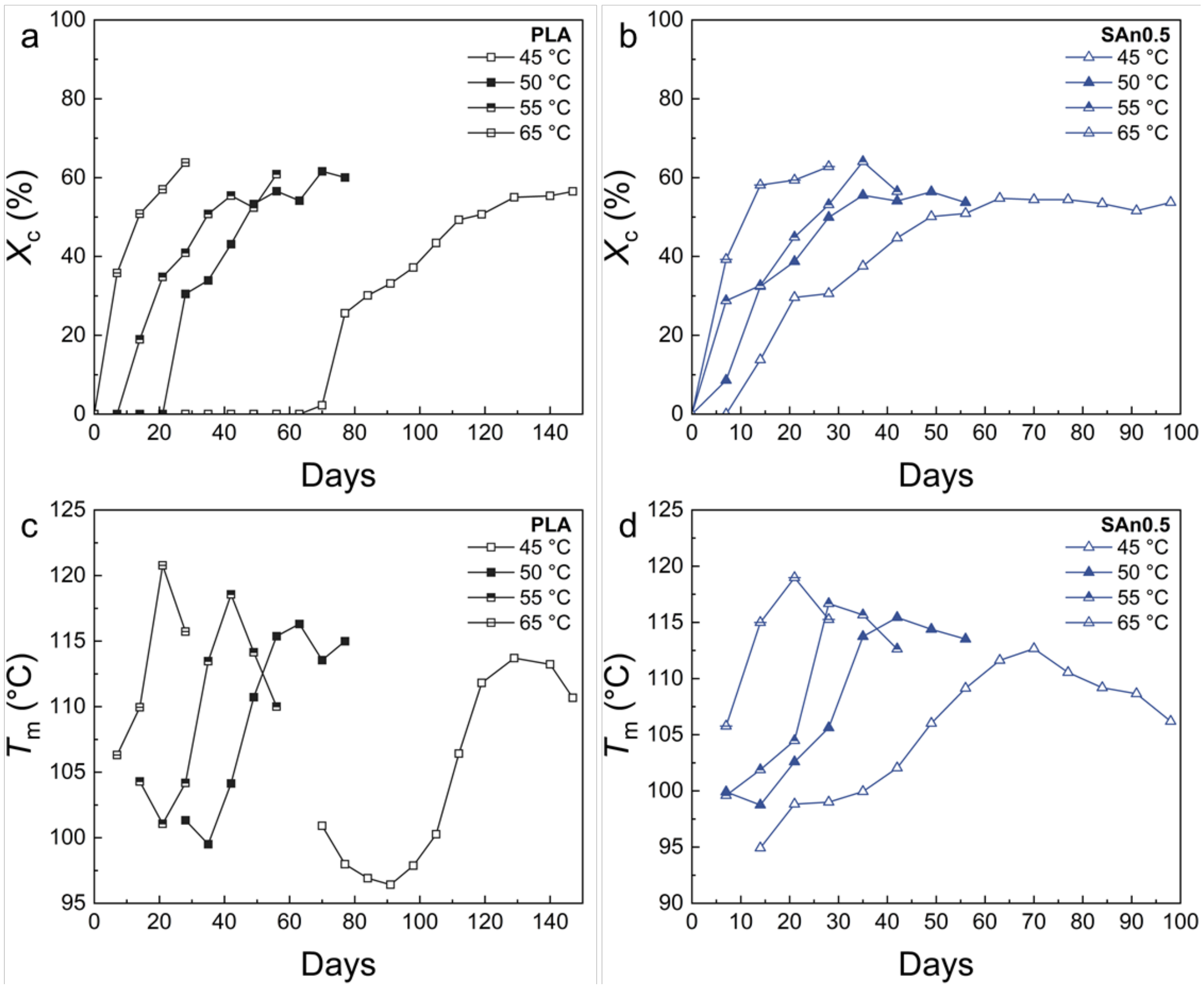

Figure S35. Crystallinity (X_c) of (a) PLA and (b) SAn0.5 and melting temperature (T_m) of (c) PLA and (d) SAn0.5 during hydrolytic degradation at various temperatures (45, 50, 55, and 65 °C) in artificial seawater. Both X_c and T_m were determined from DSC measurements.

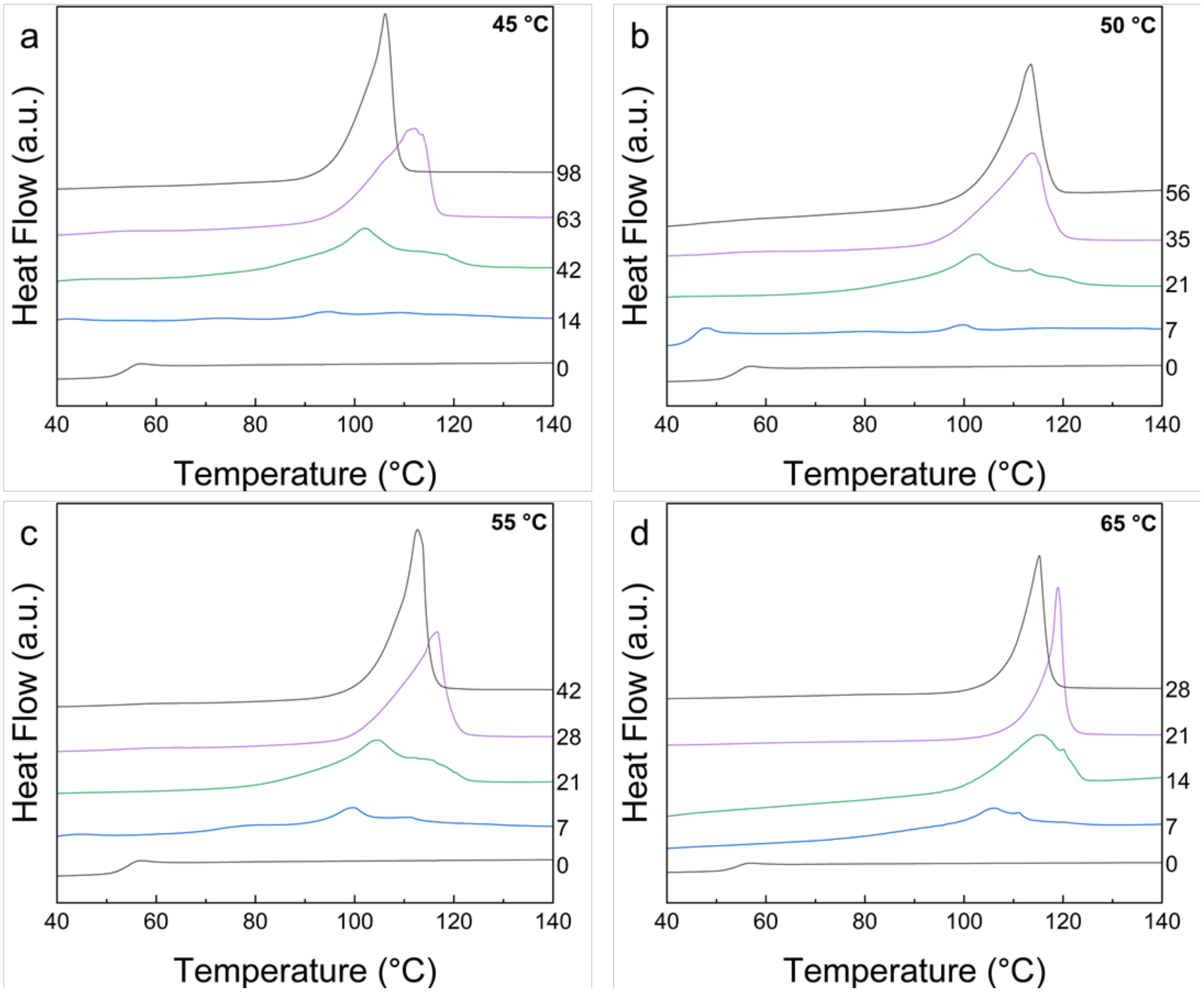

Figure S36. DSC 1st heating curves of SAn0.5 during hydrolytic degradation at (a) 45 °C, (b) 50 °C, (c) 55 °C, and (d) 65 °C. Numbers on the right indicate hydrolysis time (days).

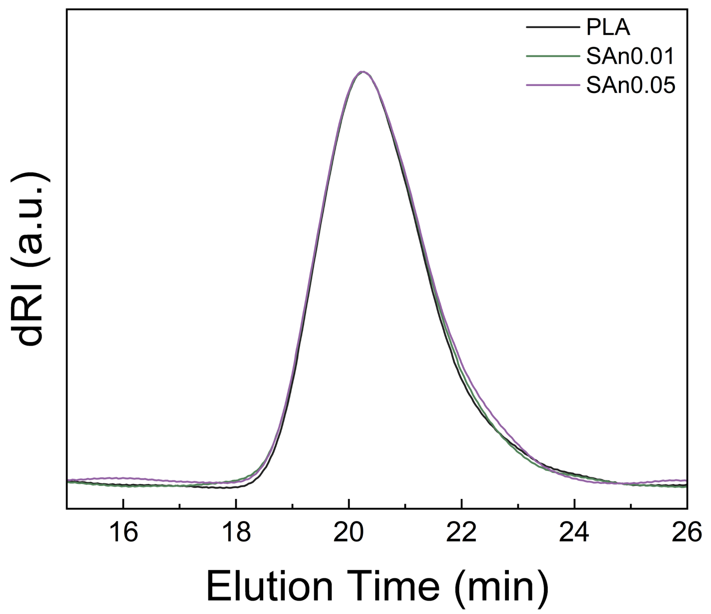

Figure S37. SEC traces of as-prepared PLA, SAn0.01, and SAn0.05 using THF as an eluent.

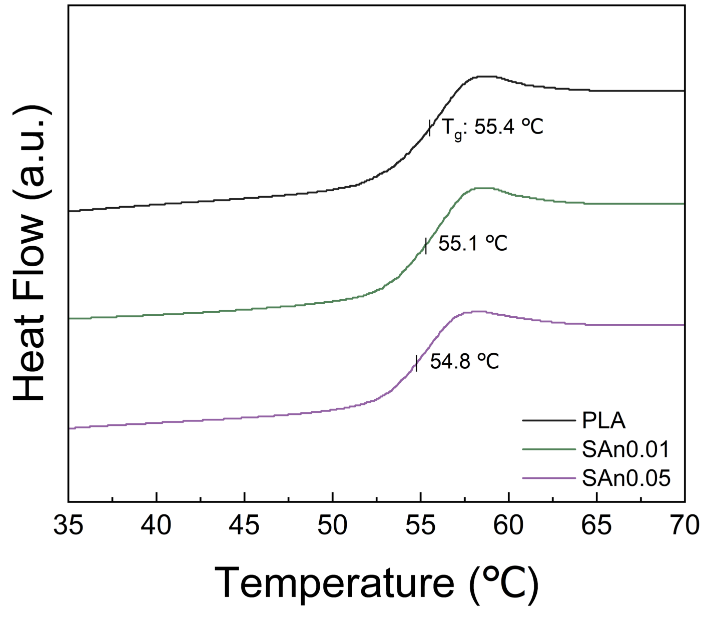

Figure S38. DSC curves (first heating, 10 °C min^{-1}) of as-prepared PLA, SAn0.01, and SAn0.05, with indicated T_g.

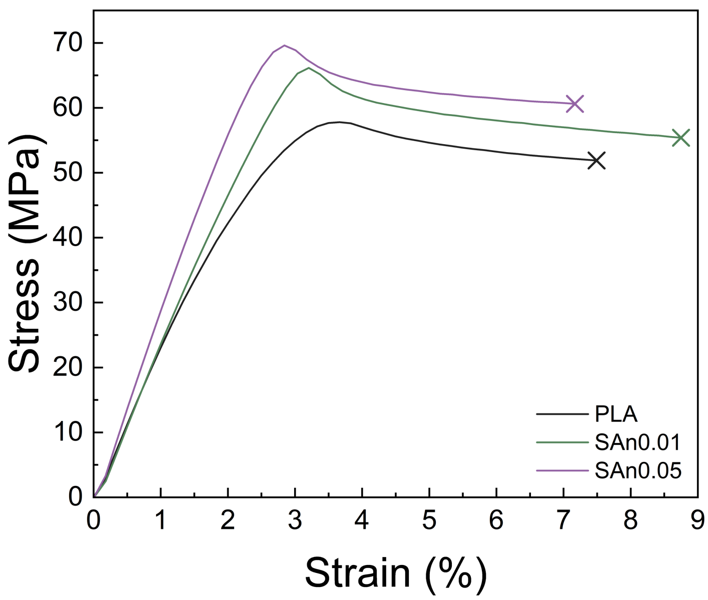

Figure S39. Representative stress-strain curves of PLA, SAn0.01, and SAn0.05 extended at 5 mm min^{-1}. The neat PLA curve is identical with that in Figure S4.

Table S7. Mechanical properties of PLA, SAn0.01 and SAn0.05.

Sample	Ultimate strength (MPa)	Young's modulus (GPa)	Strain at break (%)	Toughness (MJ m^{-3})
PLA	58.0 ± 0.6	2.5 ± 0.1	6.8 ± 2.0	3.0 ± 1.0
SAn0.01	67.6 ± 1.1	2.8 ± 0.1	8.7 ± 2.7	4.6 ± 1.6
SAn0.05	68.3 ± 1.9	3.0 ± 0.2	8.3 ± 2.9	4.3 ± 1.6

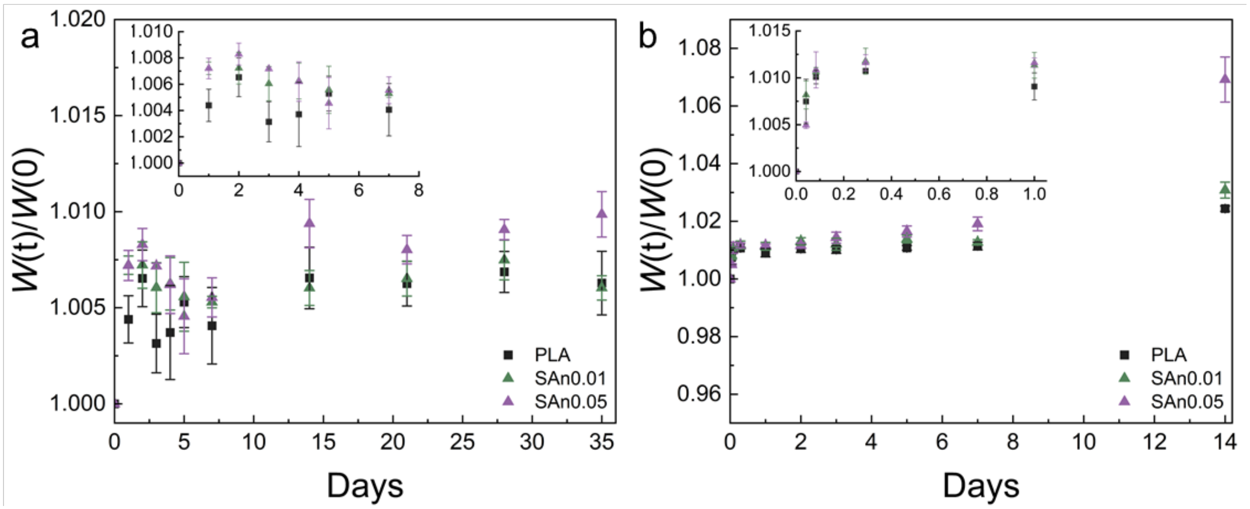

Figure S40. Normalized mass of PLA, SAn0.01, and SAn0.05 measured at (a) room temperature and (b) 50 °C under 95% relative humidity. Insets highlight the initial water uptake under each condition.

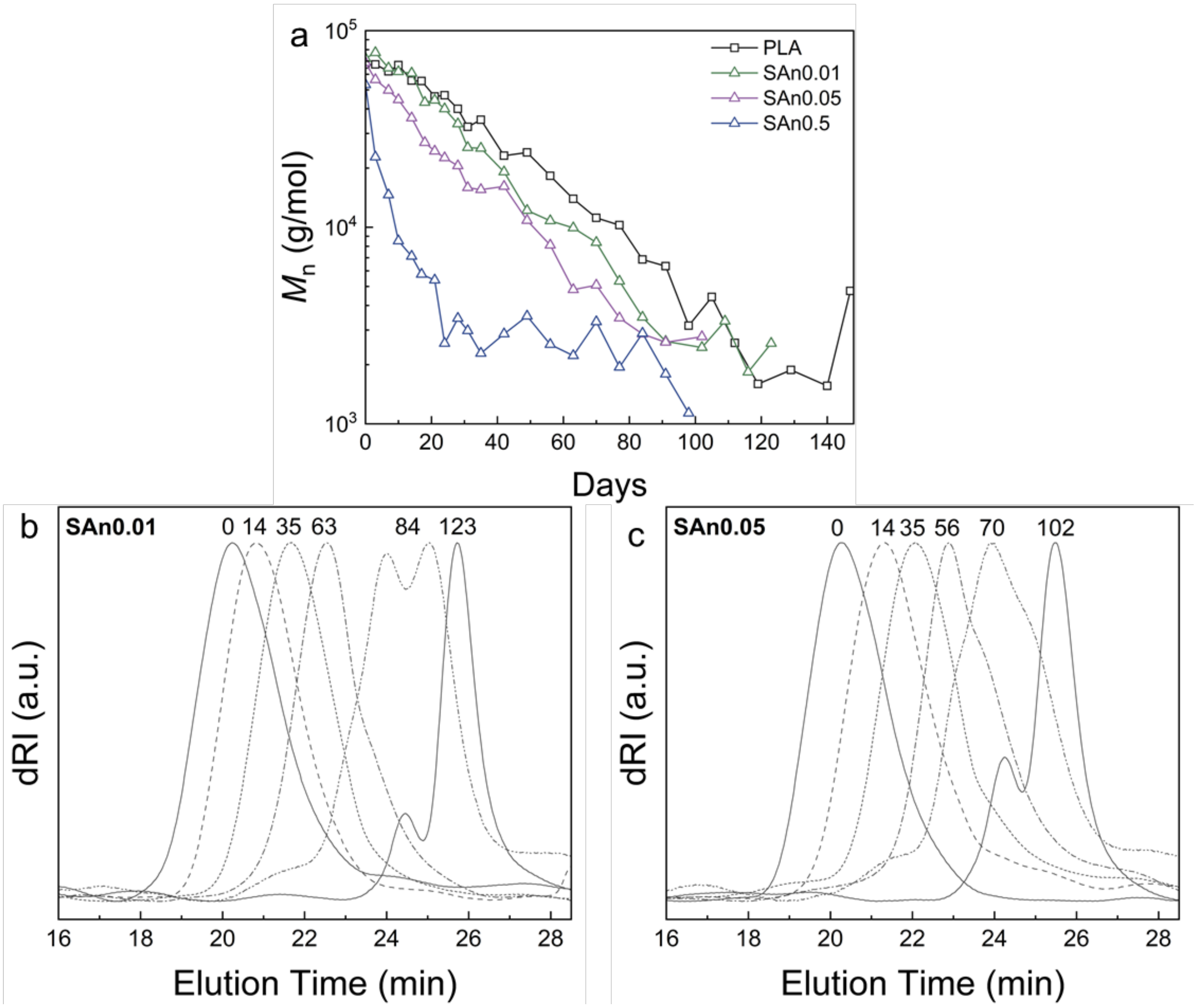

Figure S41. (a) M_n of PLA and SAn# (#: 0.01–0.5) determined from SEC results. SEC traces of (b) SAn0.01 and (c) SAn0.05 during hydrolytic degradation at 45 °C in artificial seawater. Numbers above each peak indicate the hydrolysis time (days).

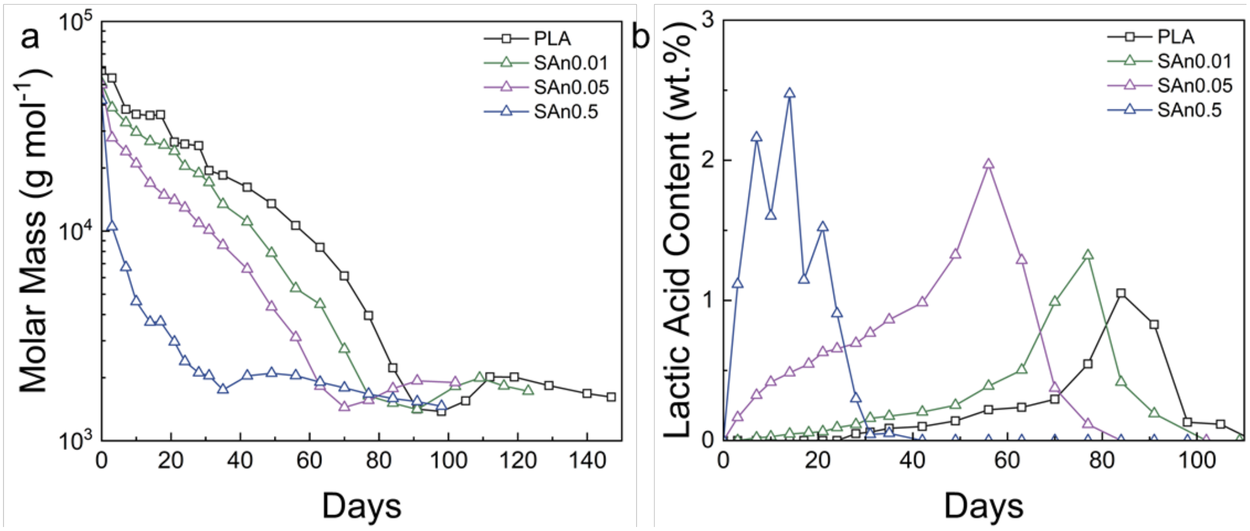

Figure S42. (a) Molar mass and (b) generated lactic acid content of neat PLA and SAn# (#: 0.01–0.5) during hydrolytic degradation at 45 °C in artificial seawater, determined using ^{1}H NMR data recorded in DMSO-d_6.

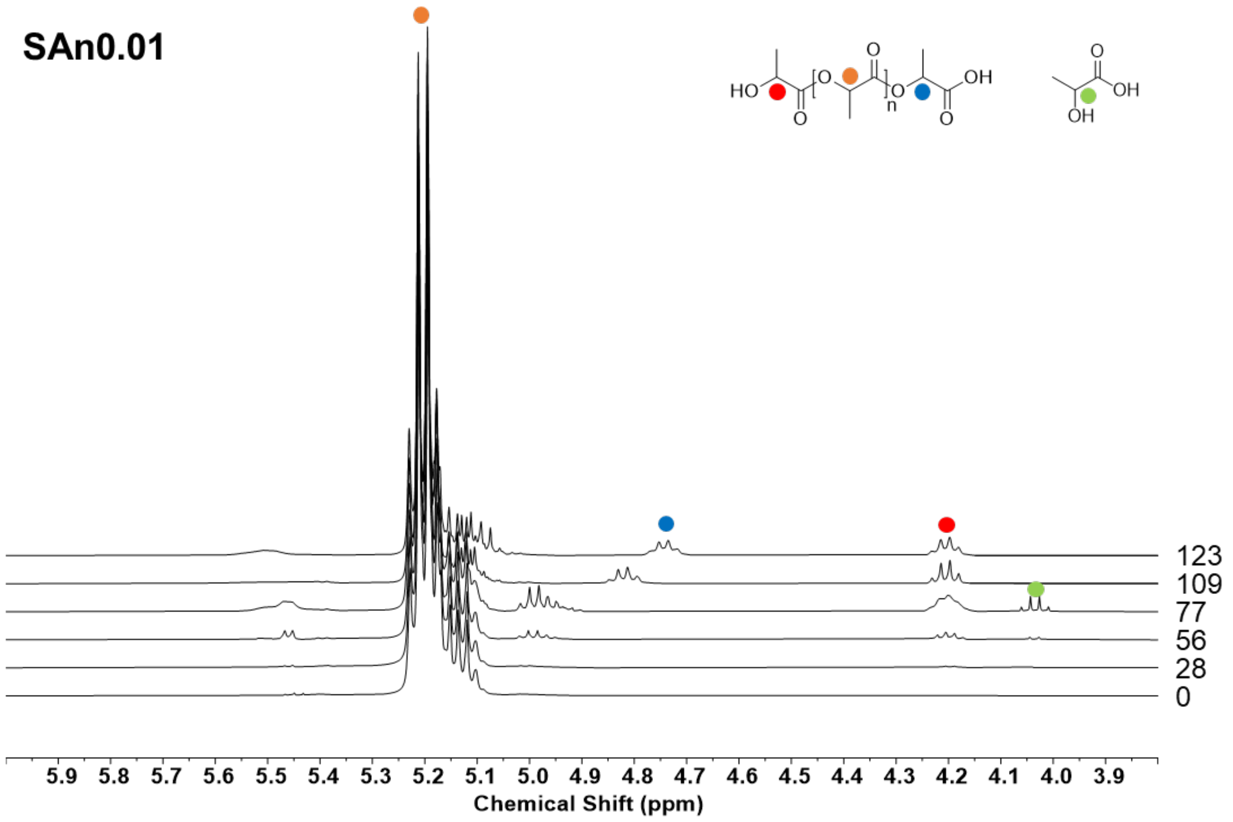

Figure S43. ^{1}H NMR spectra of SAn0.01 recorded in DMSO-d_6. Numbers on the right represent the hydrolysis time (days) in artificial seawater at 45 °C.

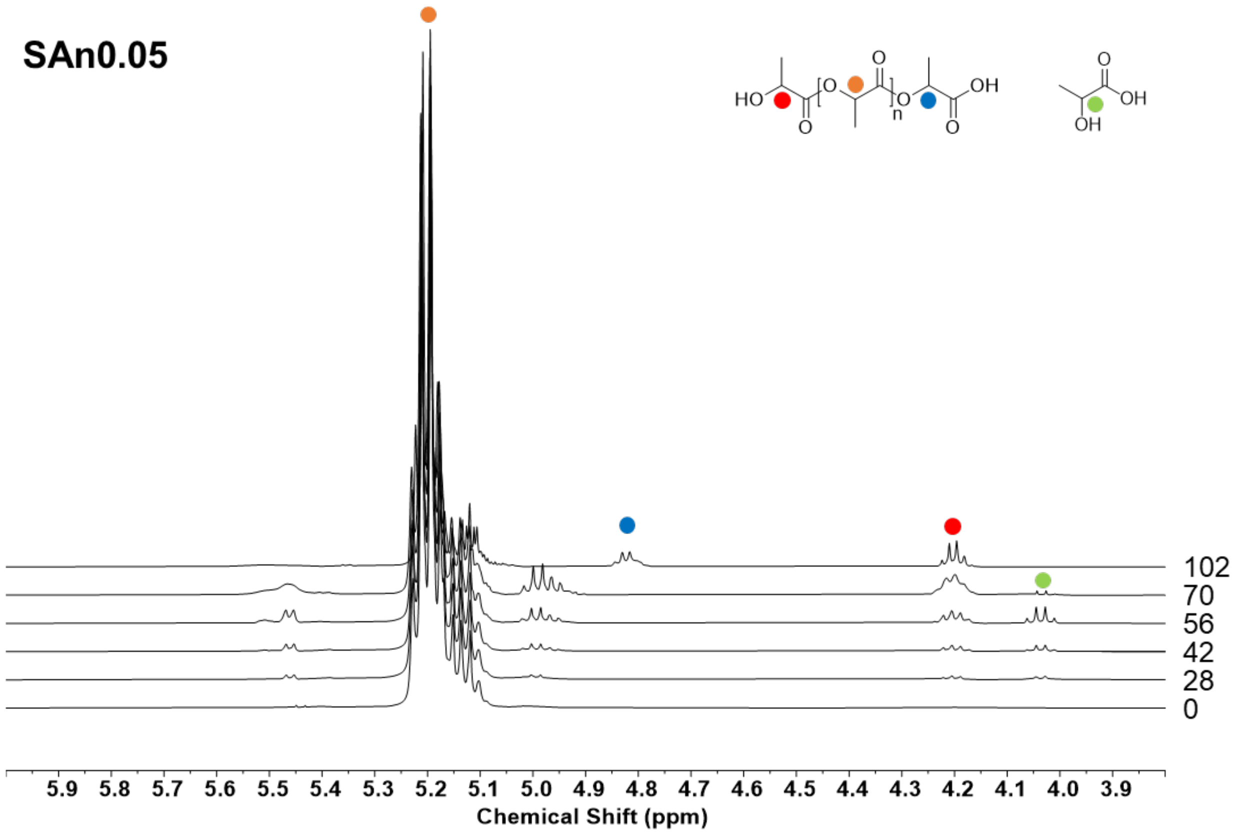

Figure S44. ^{1}H NMR spectra of SAn0.05 recorded in DMSO-d_6. Numbers on the right represent the hydrolysis time (days) in artificial seawater at 45 °C.

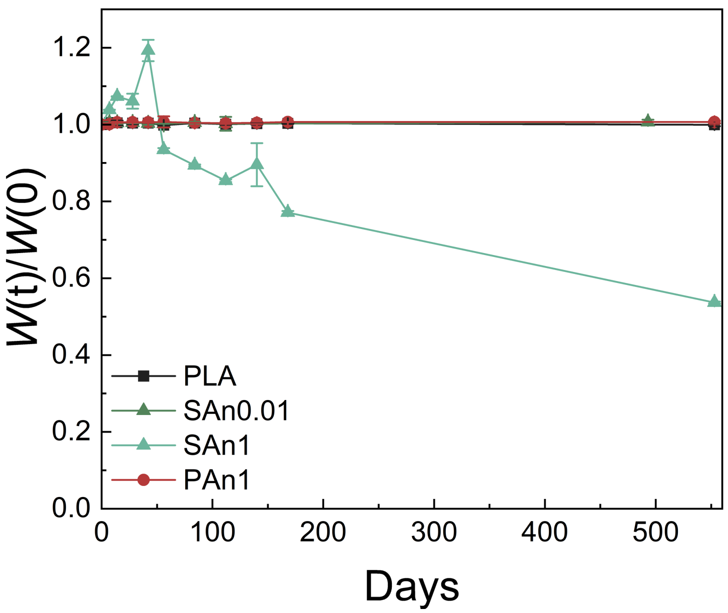

Figure S45. Fractional mass of PLA, SAn0.01, SAn1, and PAn1 during hydrolytic degradation in distilled water at ambient temperature (20–25 °C).

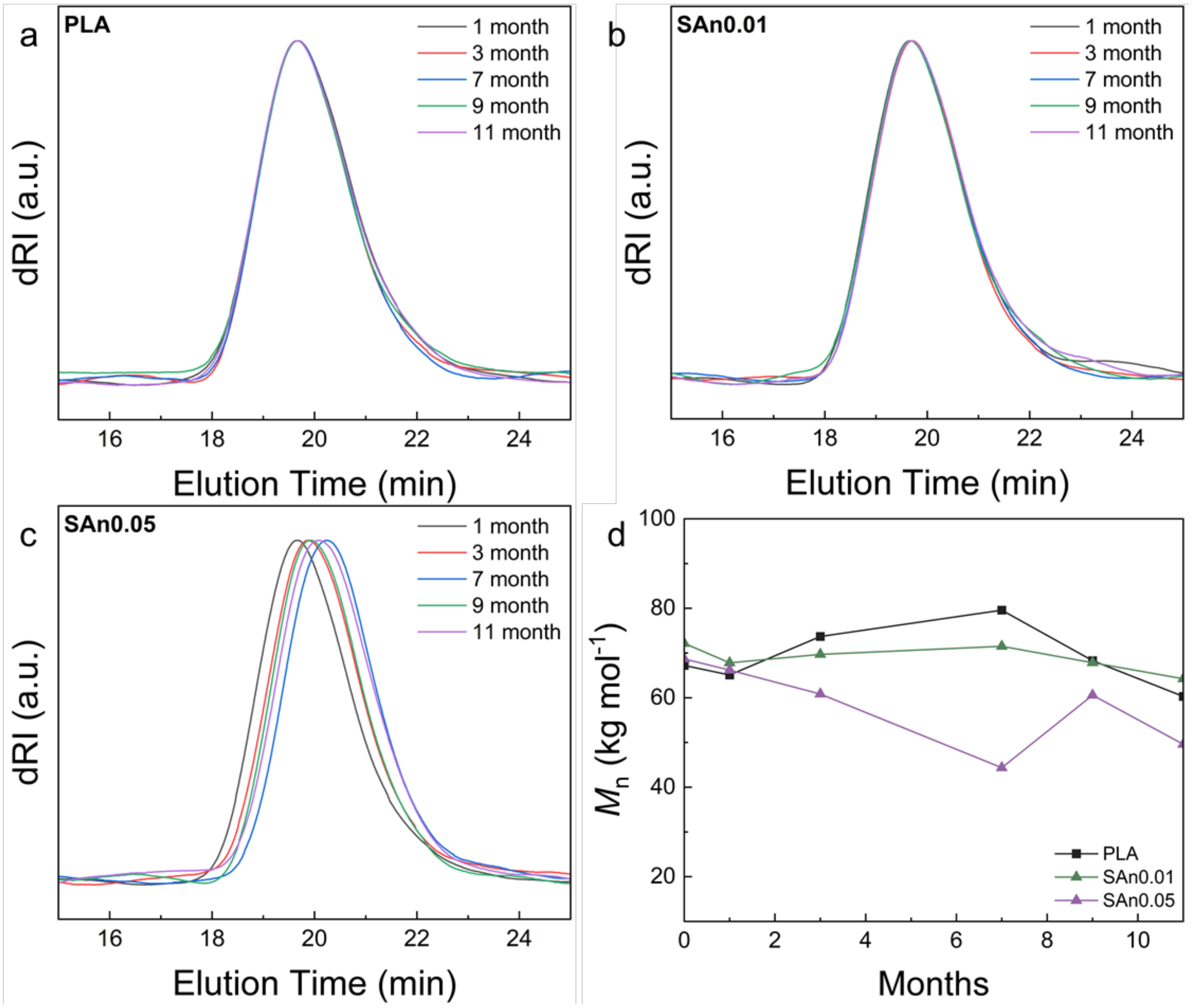

Figure S46. Representative SEC traces of (a) PLA, (b) SAn0.01, and (c) SAn0.05 stored under ambient conditions (temperature: 20–25 °C, relative humidity: 25–50%) for varying durations. (d) M_n determined from the SEC results.

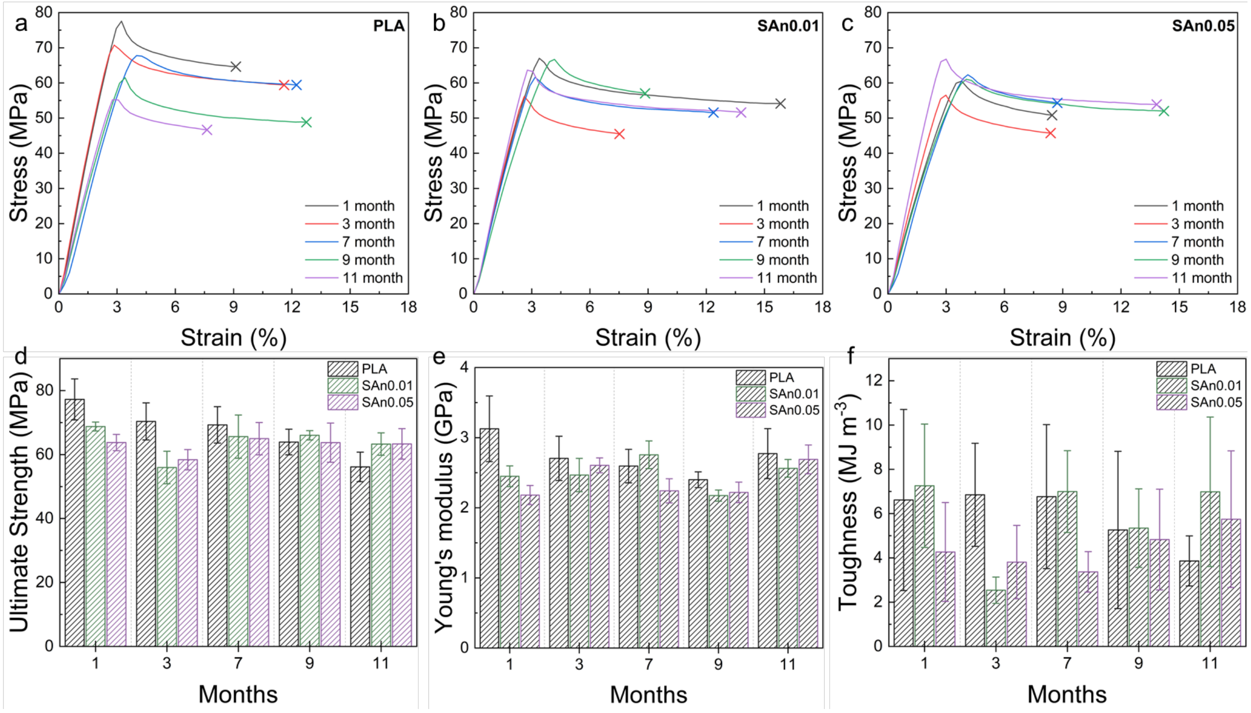

Figure S47. Representative stress-strain curves of (a) PLA, (b) SAn0.01, and (c) SAn0.05 stored under ambient conditions for varying durations. Corresponding mechanical properties derived from the tensile testing are shown for (d) ultimate strength, (e) Young's modulus, and (f) toughness. The tensile testing was conducted using dog-bone shaped specimens with dimensions 0.33 × 3.2 × 7 mm (thickness × gauge width × gauge length).

Table S8. Mechanical properties of neat PLA, SAn0.01, and SAn0.05 stored under ambient conditions for varying durations.

Samples	Ultimate strength (MPa)	Young's modulus (GPa)	Strain at break (%)	Toughness (MJ m^{-3})
PLA after 1 month	77.2 ± 6.4	3.1 ± 0.5	11.0 ± 6.1	6.6 ± 4.1
PLA after 3 months	70.4 ± 5.8	2.7 ± 0.3	12.2 ± 3.3	6.8 ± 2.3
PLA after 7 months	69.3 ± 5.7	2.6 ± 0.2	12.1 ± 4.6	6.8 ± 3.3
PLA after 9 months	63.9 ± 4.0	2.4 ± 0.1	10.8 ± 6.6	5.3 ± 3.6
PLA after 11 months	56.1 ± 4.6	2.8 ± 0.4	8.8 ± 2.0	3.9 ± 1.1
SAn0.01 after 1 month	68.8 ± 1.4	2.4 ± 0.1	13.5 ± 4.9	7.3 ± 2.8
SAn0.01 after 3 months	56.0 ± 5.1	2.5 ± 0.2	6.3 ± 1.4	2.5 ± 0.6
SAn0.01 after 7 months	65.6 ± 6.8	2.8 ± 0.2	13.3 ± 3.5	7.0 ± 1.8
SAn0.01 after 9 months	66.0 ± 1.4	2.2 ± 0.1	9.3 ± 1.7	5.3 ± 1.8
SAn0.01 after 11 months	63.3 ± 3.5	2.6 ± 0.1	13.9 ± 6.2	7.0 ± 3.4
SAn0.05 after 1 month	63.8 ± 2.5	2.2 ± 0.1	9.0 ± 3.8	4.3 ± 2.2
SAn0.05 after 3 months	58.4 ± 3.2	2.6 ± 0.1	8.6 ± 3.3	3.8 ± 1.7
SAn0.05 after 7 months	65.0 ± 5.1	2.2 ± 0.2	7.4 ± 1.8	3.4 ± 0.9
SAn0.05 after 9 months	63.7 ± 6.1	2.2 ± 0.1	11.3 ± 4.1	4.8 ± 2.3
SAn0.05 after 11 months	63.3 ± 4.8	2.7 ± 0.2	11.9 ± 6.4	5.7 ± 3.1

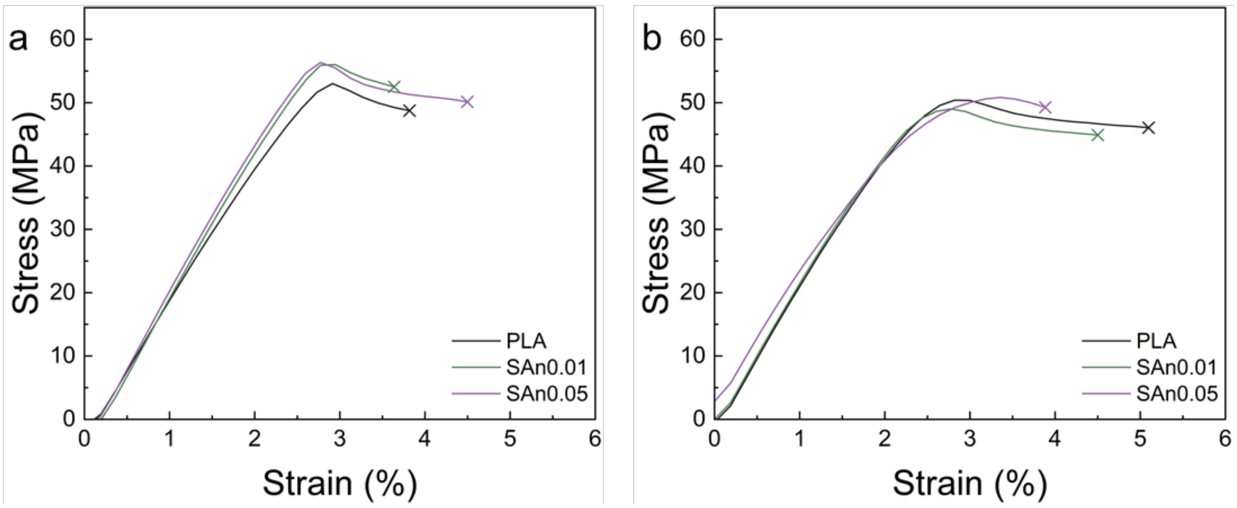

Figure S48. Representative stress-strain curves of PLA, SAn0.01, and SAn0.05 after storage under different environmental conditions: (a) ambient conditions for 3 days followed by humid conditions (temperature: 22–25 °C, relative humidity: 60–80%) for 3 days, and (b) after two repeated conditioning cycles under the same conditions.

Table S9. Mechanical properties of neat PLA, SAn0.01, and SAn0.05 subjected to cyclic conditioning, consisting of storage under the ambient conditions for 3 days followed by exposure to humid conditions for 3 days (temperature: 22–25 °C, relative humidity: 60–80%).

Samples	Ultimate strength (MPa)	Young's modulus (GPa)	Strain at break (%)	Toughness (MJ m^{-3})
PLA after 1 cycle	55.8 ± 3.1	2.5 ± 0.2	3.8 ± 0.5	1.3 ± 0.3
PLA after 2 cycles	51.8 ± 1.4	2.5 ± 0.1	5.2 ± 1.3	2.0 ± 0.6
SAn0.01 after 1 cycle	54.9 ± 1.1	2.4 ± 0.1	4.5 ± 1.3	1.7 ± 0.7
SAn0.01 after 2 cycles	50.3 ± 1.1	2.4 ± 0.1	4.6 ± 1.1	1.7 ± 0.5
SAn0.05 after 1 cycle	55.3 ± 1.0	2.5 ± 0.04	4.4 ± 1.3	1.6 ± 0.7
SAn0.05 after 2 cycles	49.9 ± 1.2	2.3 ± 0.1	4.9 ± 1.5	1.8 ± 0.7

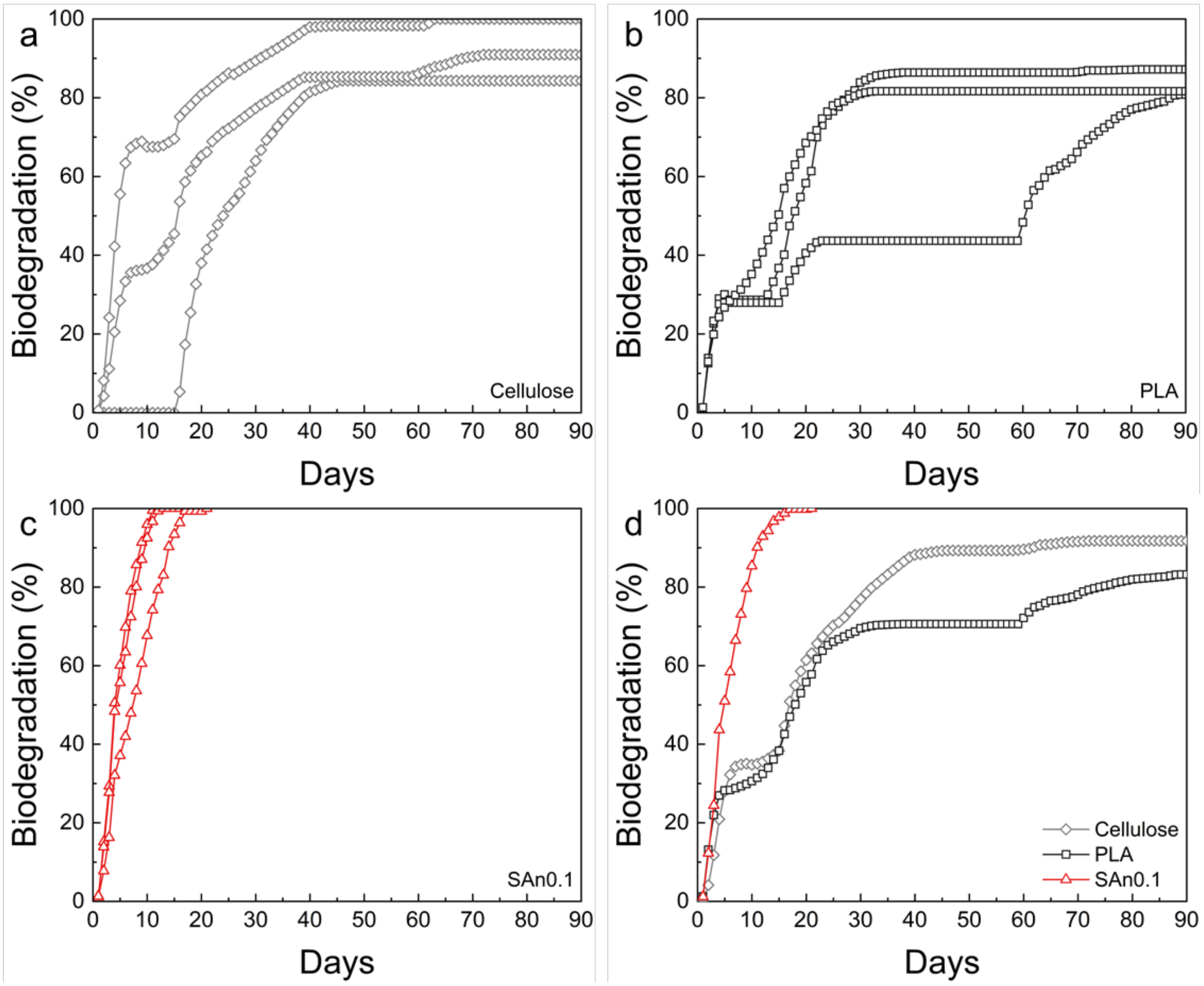

Figure S49. Absolute biodegradation profiles obtained from triplicate measurements for (a) cellulose, (b) PLA, and (c) SAn0.1. (d) Average biodegradation profiles of cellulose, PLA, and SAn0.1 calculated from the corresponding triplicates.

References

(1) ASTM International. *Standard Practice for Preparation of Substitute Ocean Water*. ASTM D1141-98; West Conshohocken, PA, 2021. DOI: 10.1520/D1141-98R21

(2) Yildirimer, L.; Seifalian, A. M. Three-dimensional biomaterial degradation - Material choice, design and extrinsic factor considerations. *Biotechnol. Adv.* **2014**, *32*, 5, 984-999.

(3) ASTM International. *Standard Test Method for Determining Aerobic Biodegradation of Plastic Materials Under Controlled Composting Conditions, Incorporating Thermophilic Temperatures*.; ASTM D5338-15; West Conshohocken, PA, 2021. DOI: 10.1520/d5338-15R21.

(4) Kalita, N. K.; Bhasney, S. M.; Kalamdhad, A.; Katiyar, V. Biodegradable kinetics and behavior of bio-based polyblends under simulated aerobic composting conditions. *J. Environ. Manage.* **2020**, *261*, 110211.

(5) Wang, L.; Ago, M.; Borghei, M.; Ishaq, A.; Papageorgiou, A. C.; Lundahl, M.; Rojas, O. J. Conductive Carbon Microfibers Derived from Wet-Spun Lignin/Nanocellulose Hydrogels. *ACS Sustainable Chem. Eng.* **2019**, *7*, 6, 6013-6022.

(6) Inkinen, S.; Hakkarainen, M.; Albertsson, A.-C.; Södergård, A. From lactic acid to poly(lactic acid) (PLA): characterization and analysis of PLA and its precursors. *Biomacromolecules* **2011**, *12*, 3, 523-532.

(7) Dhakal, H. N.; MacMullen, J.; Zhang, Z. Y. Moisture Measurement and Effects on Properties of Marine Composites. In *Marine Applications of Advanced Fibre-Reinforced Composites*; Woodhead Publishing, **2016**; pp. 103-124.

(8) Rachita, E. D.; Larison, T. S.; Hillmyer, M. A.; Ellison, C. J. Accelerated Hydrolysis of Amorphous Polylactide Containing Salicylate Additives. *ACS Sustainable Chem. Eng.* **2025**, *13*, 3, 1281-1291.
